# Reviewer acknowledgement 2015

**DOI:** 10.1186/s12889-016-2821-7

**Published:** 2016-02-25

**Authors:** Natalie Pafitis

**Affiliations:** 236 Gray’s Inn Road London, WC1X 8HB, London, UK

## Abstract

The editors of BMC Public Health would like to thank all our reviewers who have contributed to the journal in Volume 15 (2015).

**Lynette A. Hart**

USA

**Erika Aaron**

USA

**Ellen Aartun**

Canada

**Laura Aballay**

Argentina

**Fira Abamecha**

Ethiopia

**Mahdieh Abbasalizad Farhangi**

Iran

**Penelope Abbott**

Australia

**Ruzita Abd. Talib**

Malaysia

**Safa Abdalla**

USA

**Bayad Abdalrahman**

UK

**Abu Abdul-Quader**

USA

**Bayeh Abera**

Ethiopia

**Eirik Abildsnes**

Norway

**Alash'le Abimiku**

USA

**Femke Abma**

Netherlands

**Sandra Abreu**

Portugal

**Mauro Henrique Abreu**

Brazil

**Hailay Abrha**

Ethiopia

**Amina Abubakar**

Netherlands

**Laith Abu-Raddad**

Qatar

**Fikri Abu-Zidan**

United Arab Emirates

**Rajib Acharya**

India

**Chandran Achutan**

USA

**Gerardo Acosta**

Chile

**Louise Ada**

Australia

**Sunday Adaji**

Nigeria

**Jean Adams**

UK

**Bright Addo**

Ghana

**Sylvia Adebajo**

Nigeria

**Adebimpe Aderounmu**

Nigeria

**Olorunfemi Adetona**

USA

**OK Adeyemo**

Nigeria

**Getinet Adinew**

Ethiopia

**Anil Adisesh**

Canada

**Deepti Adlakha**

USA

**Karoline Aebi-Popp**

Switzerland

**Cecile Aenishaenslin**

Canada

**Olivia Affuso**

USA

**Rema Afifi**

Lebanon

**Yaw Afrane**

Kenya

**Roberta Agabio**

Italy

**Eirini Agapidaki**

Greece

**Madina Agénor**

USA

**Kingsley Agho**

Australia

**Theodoros Agorastos**

Greece

**Sutapa Agrawal**

India

**Isabel Aguilar**

Spain

**Nicolas Aguilar-Farias**

Chile

**Ojo Agunbiade**

Nigeria

**Charles Agyemang**

Netherlands

**Anand Ahankari**

UK

**Aamir Ahmad**

USA

**S.A Ahmad**

Bangladesh

**Mohamed Azmi Ahmad Hassali**

Malaysia

**Salahuddin Ahmed**

Bangladesh

**Engi Ahmed**

France

**Faruk Ahmed**

Australia

**Kedir Ahmed**

Ethiopia

**Jun Aida**

Japan

**Angela Aidala**

USA

**Hirotsugu Aiga**

Japan

**Barbara Ainsworth**

USA

**Yoshifusa Aizawa**

Japan

**Chukwuedozie Ajaero**

South Africa

**Atem Ajong**

Cameroon

**Ademola Ajuwon**

Nigeria

**Sally Akarolo-Anthony**

USA

**Manabu Akazawa**

Japan

**Sharareh Akhavan**

Sweden

**Laila Akhu-Zaheya**

Jordan

**Abiodun Akintunde**

Nigeria

**Kesiena Akpoigbe**

Canada

**Tahera Akter**

Bangladesh

**Said Al Ghora**

Palestinian Territory

**Haleama Al Sabbah**

United Arab Emirates

**Olufunke Alaba**

South Africa

**Khuder Alagha**

France

**Jane Alaii**

Kenya

**Khurshed Alam**

Bangladesh

**Arsham Alamian**

USA

**Mohammed Al-Azri**

Oman

**María-Jesús Albar**

Spain

**Steven Albert**

USA

**Julie Albrecht**

USA

**Maria de Fatima P. M. Albuquerque**

Brazil

**Maria Alcaide**

USA

**Nicol Alcina**

Brazil

**Yibeltal Alemayehu**

USA

**Laura Alfers**

South Africa

**Cristiano Alicino**

Italy

**Elias Allara**

Italy

**Stephen Allen**

UK

**Stephanie Alley**

Australia

**Amanda Allisey**

Australia

**Susannah Allison**

USA

**Kenneth Allison**

Canada

**Margaret Allman-Farinelli**

Australia

**Heather Allore**

USA

**Abdulrahman AlMohaimeed**

Saudi Arabia

**Suzana Almoosawi**

UK

**Ahmad Almulla**

Qatar

**Redhwan Al-Naggar**

Malaysia

**Ahmet Hamdi Alpaslan**

Turkey

**Khalid Al-Rasadi**

Oman

**Thamer Alshammari**

USA

**Teatske Altenburg**

Netherlands

**Pablo Altieri**

Puerto Rico

**Alejandro Alvaro-Meca**

Spain

**Alberto Alves**

Portugal

**Marcia Alves**

Brazil

**Abdulmohsen Al-Zalabani**

Saudi Arabia

**Ahmed Amara**

USA

**Alemayehu Amberbir**

Malawi

**Charles Ameh**

UK

**Daniela Amicizia**

Italy

**Tarek Amin**

Saudi Arabia

**Nayyereh Aminisani**

Australia

**Mamuda Aminu**

UK

**Noh Amit**

Malaysia

**Seth Ammerman**

USA

**Olorunfemi Amoran**

Nigeria

**Sania Amr**

USA

**Ellen Johanna Amundsen**

Norway

**Selena An**

USA

**Foteini Anastasiou**

Greece

**Gabriel Anaya**

USA

**Sophie Ancelet**

France

**Raghupathy Anchala**

India

**Alynne Andaky**

Brazil

**Ingelise Andersen**

Denmark

**Ulla Overgaard Andersen**

Denmark

**Anneka Anderson**

New Zealand

**Robert Anderson**

USA

**Alex Anderson**

USA

**Hugh Anderson**

UK

**Eileen Anderson**

USA

**Patric Andersson**

Sweden

**Laura Helena Andrade**

Brazil

**Daniela Andren**

Sweden

**Martin Andresen**

Canada

**Aranka Anema**

USA

**Michael Anestis**

USA

**Daniel Ankrah**

Ghana

**Matilda Annerstedt**

Sweden

**Nana Anokye**

UK

**Y. Gavriel Ansara**

Australia

**Rashid Ansumana**

Sierra Leone

**Peter Anthamatten**

USA

**Denis Anthony**

UK

**Jose Leopoldo Ferreira Antunes**

Brazil

**Adote Anum**

Ghana

**R Anuradha**

India

**Liu Ap**

China

**John Apolzan**

USA

**Joana Araújo**

Portugal

**Jayashree Arcot**

Australia

**Chris Ardern**

Canada

**Maria Argos**

USA

**Maria Angeles Argudin**

Belgium

**Elva Arias-Merino**

Mexico

**Ayman Arif**

Jordan

**Shams Arifeen**

Bangladesh

**Hisatomi Arima**

Japan

**Nicolas Arnaud**

Germany

**Sigurbjörn Arngrímsson**

Iceland

**Arja R Aro**

Denmark

**Robert Aronson**

USA

**Rashmi Arora**

India

**Bonnie Arquilla**

USA

**Britt Arrelov**

Sweden

**Silvina Arrossi**

Argentina

**Kwaku Poku Asante**

Ghana

**Richard Ashcroft**

UK

**Ian Askew**

Kenya

**Parisa Aslani**

Australia

**Felix Assah**

Cameroon

**Shervin Assari**

USA

**Nega Assefa**

Ethiopia

**Mulubirhan Assefa**

Ethiopia

**Maria Athanasiou**

Greece

**Vasilios Athyros**

Greece

**Andrew J Atkin**

UK

**Robert Atkins**

USA

**Sachin Atre**

India

**Michael Attfield**

USA

**Lukoye Atwoli**

Kenya

**Carolyn Audet**

USA

**Suzanne Audrey**

UK

**Colette Auerswald**

USA

**Lana Augustincic**

Canada

**Dagfinn Aune**

UK

**Beth Auslander**

USA

**Birgit Aust**

Denmark

**Aine Aventin**

UK

**Abdelmoneim Awad**

Kuwait

**Rukman Awang Hamat**

Malaysia

**Olutosin Alaba Awolude**

Nigeria

**Irene Ayakaka**

Uganda

**Andrew Azman**

USA

**Netsanet Fentahun Babbel**

Ethiopia

**Hannah Badland**

Australia

**Anne-Marie Bagnall**

UK

**Teresa Bago d'Uva**

Netherlands

**James Bagonza**

Uganda

**Ross Bailie**

Australia

**Mohan Bairwa**

India

**Anna Bajer**

Poland

**Taoufik Bakkali**

India

**Inger Johanne Bakken**

Norway

**Frances Balamuth**

USA

**Bhuvaneswari Balan**

India

**Hector Balcazar**

USA

**Alberto Baldasseroni**

Italy

**Vincenzo Baldo**

Italy

**Tatjana Baldovin**

Italy

**Carolina Ballert**

Switzerland

**Mobolanle Balogun**

Nigeria

**Eric Balti**

Belgium

**Tara Singh Bam**

Nepal

**Dirgh Singh Bam**

Nepal

**Mike Bambach**

Australia

**Matthew Bambling**

Australia

**Hilary Bambrick**

Australia

**Sudip Banik**

Mexico

**Dharanidhar Baral**

Nepal

**Jed Barash**

USA

**Mary Barbe**

USA

**Shaun Barber**

UK

**Gerson Barbosa**

Brazil

**Russell Barbour**

USA

**Kieron Barclay**

Sweden

**Anamitra Barik**

India

**Salesa Barja**

Chile

**Kris Barnekow**

USA

**Elizabeth Barnett**

USA

**Karen Barnett**

UK

**Margo Barr**

Australia

**Pamela Barrios**

USA

**Pamela Barrios**

USA

**Xavier Bartoll**

Spain

**Robert Basaza**

Uganda

**Diego Bassani**

Canada

**Sandra Bassett**

New Zealand

**Anwar Batieha**

Jordan

**Annette Bauer**

UK

**Heidi Bauer**

USA

**Shawn Bauldry**

USA

**Carl Baum**

USA

**Michele Baumann**

Luxembourg

**Sophie Baumann**

Germany

**Daniel Bausch**

USA

**Henry Bautista-Amorocho**

Colombia

**Amanda J Baxter**

Australia

**Adam Baxter-Jones**

Canada

**Antony Bayer**

UK

**Jude Bayham**

USA

**Alexander Bazazi**

USA

**Stefania Bazzo**

Italy

**Alison Beauchamp**

Australia

**Stevens Bechange**

Uganda

**Angela Bechini**

Italy

**Bernard Beckerman**

USA

**Richard Bedell**

Canada

**Malgorzata Bednarska**

Poland

**Giorgio Bedogni**

Italy

**Karlyn Beer**

USA

**Stephen Begg**

Australia

**Ulla Beijer**

Sweden

**Elaine Belansky**

USA

**Mulugeta Belay**

Ethiopia

**Andrew Bell**

UK

**Ruth Bell**

UK

**Robin Bell**

Australia

**Folasade Bello**

Nigeria

**Silvia Bel-Serrat**

Spain

**Hiram Beltrán-Sánchez**

USA

**Cristiane Bendo**

Brazil

**Sara E Benjamin Neelon**

USA

**Abdelouaheb Bennani**

Morocco

**Jeanette Bennett**

USA

**Jason Bennie**

Australia

**T. Benson**

UK

**Michael Bentley**

Australia

**Rebecca Bentley**

Australia

**Joan Bentzen**

Denmark

**David Beran**

Switzerland

**Janneke Berecki-Gisolf**

Australia

**Karianne Berg**

Norway

**Roos Maria Desirée Bernsen**

Netherlands

**Sveinung Berntsen**

Norway

**David Berrigan**

USA

**Jane Bertrand**

USA

**Aniella Beser**

Sweden

**Jeffrey Bethel**

USA

**Matthias Bethge**

Germany

**Nicholas Beyler**

USA

**Woldesellassie Bezabhe**

Australia

**Arvin Bhana**

South Africa

**S Bhandare**

India

**Miteshkumar Bhanderi**

India

**Rajni Bharati**

Nepal

**Bhavneet Bharti**

India

**Krishnan Bhaskaran**

UK

**Jatinder Bhatia**

USA

**Shinjini Bhatnagar**

India

**Junaid Bhatti**

Canada

**Raj Bhopal**

UK

**Silvia Bianchi**

Italy

**Satesh Bidaisee**

Grenada

**Stuart Biddle**

Australia

**Robin Biellik**

UK

**Godfrey Biemba**

Zambia

**Simona Bignami**

Canada

**H Bihan**

France

**Benjamin T. Bikman**

USA

**Monica Biradavolu**

USA

**Tannaz Birdi**

India

**Dereje Birhanu**

Ethiopia

**Michelle Birkett**

USA

**Caroline Biron**

Canada

**Alexander Bischoff**

Switzerland

**Zeno Bisoffi**

Italy

**Cyrille BIsseye**

Gabon

**Renee Bittoun**

Australia

**Vesna Bjegovic-Mikanovic**

Serbia

**Mona Bjelland**

Norway

**Kirsten Black**

Australia

**Nancy Black**

Canada

**Stuart Blacksell**

Thailand

**Alex Blaszczynski**

Australia

**Sergio Blay**

Brazil

**Jonathan Blitstein**

USA

**Stephanie Block**

USA

**Michael Bloom**

USA

**Ricky Bluthenthal**

USA

**Helen Boardman**

UK

**Jason Bocarro**

USA

**Sara Boccalini**

Italy

**Delia Boccia**

UK

**Daniela Boccolini**

Italy

**Petri Böckerman**

Finland

**Owen Bodger**

UK

**Julia Boehm**

USA

**Olga Bogoliubova**

Russian Federation

**Laura Bojke**

UK

**Cecilia Boldemann**

Sweded

**Guglielmo Bonaccorsi**

Italy

**Chris Bonell**

UK

**Billie Bonevski**

Australia

**Matteo Bonzini**

Italy

**Matthias Bopp**

Switzerland

**Jacob Bor**

USA

**Johan Borg**

Sweden

**Michael Borghese**

Canada

**Daniel Bornstein**

USA

**Judit Bort-Roig**

Spain

**Judith Bos**

Netherlands

**Colin Bos**

Netherlands

**Marije Bosch**

Australia

**Julitta Boschman**

Netherlands

**Francis Boscoe**

Bouvet Island

**Hans Bosma**

Netherlands

**Wendy Bostwick**

USA

**Matthys Botha**

South Africa

**Nectarios Boubopoulos**

Greece

**Soufiane Boufous**

Australia

**Stephen Boutcher**

Australia

**Angela Bowen**

Canada

**Erick Boy**

Canada

**Marek Brabec**

Czech Republic

**Ylisabyth Bradshaw**

USA

**Nicola Luigi Bragazzi**

Italy

**Nicola Bragazzi**

Italy

**Jennifer Bragg-Gresham**

USA

**Julii Brainard**

UK

**Paula Braitstein**

USA

**Tilman Brand**

Germany

**Rachel Brathwaite**

UK

**Alexandra Brazinova**

Austria

**Jeff Breckon**

UK

**João Breda**

Denmark

**Elizabeth Breeze**

UK

**Joshua Breslau**

USA

**Christian Brettschneider**

Germany

**Andre Briend**

USA

**Julie Brimblecombe**

Australia

**Lucy Brindle**

UK

**Yolandi Brink**

South Africa

**Jason Brinkley**

USA

**Wendy Brodribb**

Australia

**Carinne Brody**

USA

**Elizabeth Brondolo**

USA

**Liza Bronner Murrison**

USA

**Hannah Brooke**

UK

**Katie Brooker**

Australia

**Pierre Brouard**

South Africa

**Amanda Brouwer**

USA

**Rachel Brown**

New Zealand

**Jac Brown**

Australia

**Helen Brown**

Australia

**Sally Brown**

UK

**Tamara Brown**

UK

**Stephanie Broyles**

USA

**Roberto Bruni**

Italy

**Silvio Brusaferro**

Italy

**Johan Nikolai Bruun**

Norway

**Dag Bruusgaard**

Norway

**Robert Brychta**

USA

**David Buchanan**

USA

**Jeanine Buchanich**

USA

**Susan Buchholz**

USA

**Maciej Buchowski**

USA

**Christoph Buck**

Germany

**Jens Bucksch**

Germany

**Lygia Therese Budnik**

Germany

**Merete Drevvatne Bugge**

Norway

**Dominic Bukenya Yiga**

Uganda

**Craig Burgess**

Vietnam

**Miria Burgos**

Brazil

**Rachel Burke**

USA

**Jessica Burke**

USA

**Sharyn Burns**

Australia

**Carol Burns**

USA

**Sharyn Burns**

Australia

**Hermann Burr**

Germany

**Tracy Burrows**

Australia

**Igor Burstyn**

USA

**Marta Cecilia Busana**

UK

**Marta Busana**

UK

**Luca Busani**

Italy

**Susan Buskin**

USA

**Eduardo Bustamante**

USA

**Alexander Butchart**

Switzerland

**Lisa Butler**

USA

**Bilal Butt**

USA

**Gabriele Buttinelli**

Italy

**Mary Katepa Bwalya**

Zambia

**Peter Byass**

Sweden

**Stine Byberg**

Denmark

**Theresa Byrd**

USA

**Carol Byrd-Bredbenner**

USA

**Thomas Byrne**

USA

**Francisco Félix Caballero**

Spain

**Noriko Cable**

UK

**Yann Cabon**

France

**Esther Cabrera**

Spain

**Dorina Cadar**

UK

**Cristina Cadenas-Sánchez**

Spain

**Louise Cadman**

UK

**Lisa Cadmus-Bertram**

USA

**Ennio Cadum**

Italy

**Kathryn Cairns**

Australia

**Natalia Calanzani**

UK

**Alison Calear**

Australia

**Emily Callander**

Australia

**Denton Callander**

Australia

**Peter Callery**

UK

**Lidyane Camelo**

Brazil

**Adrian Cameron**

Australia

**Christine Cameron**

Canada

**Daniel Camiletti-Moirón**

Spain

**Adriana Campa**

USA

**Ahmet Selçuk Can**

Turkey

**Jessica Cance**

USA

**Math Candel**

Netherlands

**Rosa Cano**

Spain

**Dexter Canoy**

UK

**Zhidong Cao**

China

**Chunxiang Cao**

China

**D Capron**

USA

**Martin Caraher**

UK

**Gemma Carey**

Australia

**Shannon Carlin-Menter**

USA

**Silvia Carlos**

Spain

**Axel Carlsson**

Sweden

**Troy Carlton**

USA

**Nancy Carnide**

Canada

**Margherita Caroli**

Italy

**Christin Carotta**

USA

**Pilar Carrasco-Garrido**

Spain

**Rodrigo Carrillo**

Peru

**Kristin Carson**

Australia

**Owen Carter**

Australia

**Eric Carter**

USA

**Michele Carugno**

Italy

**Rocio Casanas**

Spain

**Patricia Casey**

Ireland

**Annalisa Casini**

Belgium

**Ann Cassady**

USA

**Tali Cassify**

USA

**J. David Cassidy**

Canada

**Carlos Castañeda-Orjuela**

Colombia

**Carmen Castaneda-Sceppa**

USA

**Francesco Castelli**

Italy

**Lilia Castillo-Martinez**

Mexico

**Erika Castro**

Switzerland

**Enrique Castro-Sánchez**

UK

**Janine Cataldo**

USA

**Sheryl Cates**

USA

**David Cavallo**

USA

**Nick Cavill**

UK

**Giulia Cesaroni**

Italy

**Anna Chaimani**

Greece

**Venkatesan Chakrapani**

India

**Kirsty Challen**

UK

**Christina Chambers**

USA

**Jane Champion**

USA

**Heng Choon (Oliver) Chan**

Hong Kong

**Pascalina Chanda-Kapata**

Zambia

**Geetanjali Chander**

USA

**Daniel Chandramohan**

UK

**Kiara Chang**

UK

**Chih-Cheng Chang**

Taiwan

**I-Shou Chang**

Taiwan

**Paul Chantler**

USA

**Gwen Chapman**

UK

**Katrina Charles**

UK

**André Charlett**

UK

**Sebastien Chastin**

UK

**Josephine Chau**

Australia

**Kee Chee Cheong**

Malaysia

**Daniel Chemtob**

Israel

**Brian Chen**

USA

**Jie Chen**

USA

**Renjie Chen**

China

**Wan Qing Chen**

China

**Yiyn Chen**

China

**Lei-Shih Chen**

USA

**Shu-Ping Chen**

Canada

**Chu-Chih Chen**

Taiwan

**Feon Cheng**

USA

**Cecilia Cheng**

Hong Kong

**Man Ying Edith Cheng**

UK

**Haw-Kwan Cheong**

Korea

**Nicola Cherry**

Canada

**Doug Cheung**

USA

**Peter Cheung**

Singapore

**Peilian Chi**

Macao

**Guangqing Chi**

USA

**Alain Chichom**

Cameroon

**Ling-Chu Chien**

Taiwan

**Violet Chihota**

South Africa

**Antony Chikutsa**

Zimbabwe

**Palma Chillón**

Spain

**Mariana Chilton**

USA

**LA Chimoyi**

South Africa

**Tobias Chirwa**

South Africa

**Nathaniel Chishinga**

Zambia

**Jack Chisum**

USA

**Simbarashe Chitanga**

Zambia

**Ya-Wen Chiu**

Taiwan

**Young Cho**

USA

**BongKyoo Choi**

USA

**Eunhee Choi**

USA

**Jeongjoon Choi**

USA

**Hojoon Choi**

USA

**M Choisy**

USA

**Herberto Jose Chong Neto**

Brazil

**Kee-Lee Chou**

China

**Vikas Choudhry**

Sweden

**Eric Chow**

Australia

**Hayley Christian**

Australia

**Nicola Christie**

UK

**Celia Dana Claire Christie-Samuels**

Jamaica

**Anthea Christoforou**

Canada

**Jonathan Chua**

Singapore

**Kenneth Chui**

USA

**Jae Hoon Chung**

South Korea

**Benjamin Chung**

USA

**Grace Chung**

USA

**Bruno Christian Ciancio**

Sweden

**Fabio Cibella**

Italy

**Anna Rita Ciccaglione**

Italy

**Dana Karen Ciccone**

USA

**Nursan Cinar**

Turkey

**Maria Grazia Ciufolini**

Italy

**Giovanni cizza**

USA

**Mora Claramita**

Indonesia

**Bronwyn Clark**

Australia

**Thomas Clasen**

UK

**Cristina Cleghorn**

New Zealand

**John Cleland**

UK

**Timo Clemens**

Netherlands

**Christopher John Clements**

Australia

**Svea Closser**

USA

**Alan Clough**

Australia

**Sean Clouston**

USA

**Lucie Cluver**

UK

**Lynn Cockburn**

Canada

**Anne Cockcroft**

Botswana

**Christopher Codispoti**

USA

**Manuel Coelho e Silva**

Portugal

**Steven Cohen**

USA

**Craig Cohen**

USA

**Carlos E. A. Coimbra Jr.**

Brazil

**Jan Coles**

Australia

**Paul Collings**

UK

**Harry Comber**

Ireland

**Yvonne Commodore-Mensah**

USA

**Elizabeth Comrie-Thomson**

Australia

**Emelie Condén**

Sweden

**Katherine Conigrave**

Australia

**Eric Connolly**

USA

**Avonne Connor**

USA

**Deborah Constant**

South Africa

**Phoebe Constantinou**

USA

**Lisa Conti**

USA

**Gabriella Conti**

UK

**Jackie Cook**

UK

**Ian Cook**

South Africa

**Martin Cooke**

Canada

**Kerri Coomber**

Australia

**Robert Cooney**

USA

**Peter Cooper**

South Africa

**Kumarasen Cooper**

USA

**Robert Copeland**

UK

**Robert Copeland**

UK

**R Coqueiro**

Brazil

**Jason Corburn**

USA

**Rhiannon Corcoran**

UK

**Kirsten Corder**

UK

**Cesare Cornaggia**

Italy

**Bert Cornelius**

Netherlands

**Stephen M. Cornish**

Canada

**Eva Corpeleijn**

Netherlands

**Patricia Correa-Faria**

Brazil

**Karen Corsi**

USA

**Phaedra Corso**

USA

**Silvia Costa**

UK

**Giorgio Costantino**

Italy

**Ryan Courtney**

Australia

**Kimberly Cousins**

New Zealand

**Gill Cowburn**

UK

**Georgina Cox**

Australia

**Doina Cozman**

Romania

**Susanna Cramb**

Australia

**AliceAnn Crandall**

USA

**Jo Cranwell**

UK

**Douglas Crawford-Brown**

UK

**Fiona Crawford-Williams**

Australia

**Hilary Creed-Kanashiro**

Peru

**Paul Cremers**

Netherlands

**Aidan Cronin**

Indonesia

**Trevor Crowell**

USA

**Tania Crucitti**

Belgium

**Rik Crutzen**

Netherlands

**Salvador Cruz-Flores**

USA

**Ilona Csizmadi**

Canada

**Sharon Cummins**

USA

**Joana Cunha-Cruz**

USA

**Solveig Cunningham**

USA

**Leslie Cunningham-Sabo**

USA

**Barbara Curbow**

USA

**Douglas Curran-Everett**

USA

**Whitney B. Curry**

UK

**Carmen Cuthbertson**

USA

**Chantal Daas**

Netherlands

**Peter D'Abbs**

Australia

**Berihun Dachew**

Ethiopia

**Rada Dagher**

USA

**Darren Dahly**

Ireland

**Luigino Dal Maso**

Italy

**Ann Marie Dale**

USA

**Amanda Daley**

UK

**Peter Daley**

Canada

**Timothy Dall**

USA

**Anne Daltveit**

Norway

**Elizabeth D'Amico**

USA

**Oliver Damm**

Germany

**Kelsey Dancause**

Canada

**Kelsey Dancause**

Canada

**Fortunato D'Ancona**

Italy

**Rakhi Dandona**

India

**Alan Dangour**

UK

**Joseph Daniels**

USA

**Tor Erik Danielsen**

Norway

**Andrew Dannenberg**

USA

**M. Carolina Danovaro-Holliday**

USA

**An Dao**

Vietnam

**Nihaya Daoud**

Israel

**Shrinivas Darak**

India

**Lynae Darbes**

USA

**Carl D'Arcy**

Canada

**Frances Dark**

Australia

**Catherine Darker**

Ireland

**Magdy Darwish**

Saudi Arabia

**Jai Das**

Pakistan

**Undurti Das**

USA

**Jayati Das-Munshi**

UK

**Daniel Datiko**

Ethiopia

**Mike Daube**

Australia

**Bege Dauda**

Belgium

**Asli Davas**

Turkey

**Amy Davidow**

USA

**Leslie L. Davidson**

USA

**Cally Davies**

Canada

**Ian Davies**

UK

**Teresa Davis**

Australia

**James Davis**

USA

**Mark Davis**

Australia

**Adrian Davis**

UK

**Kirsten Davison**

USA

**Karen Davison**

Canada

**Martin Davoren**

Ireland

**Brenda Davy**

USA

**Halima Dawood**

South Africa

**Eling D. de Bruin**

Switzerland

**Katrien De Cocker**

Belgium

**Hannelore De Grande**

Belgium

**Emilia I. De la Fuente**

Spain

**Dylan de Lange**

Netherlands

**Evelyne de Leeuw**

Australia

**Tim De Maayer**

South Africa

**Dario De Medici**

Italy

**Pilar De Miguel-Etayo**

Spain

**Cesar De Oliveira**

UK

**Angelique de Rijk**

Netherlands

**Susanne de Rooij**

Netherlands

**Delphine De Smedt**

Belgium

**Anniza De Villiers**

South Africa

**Sanne de Vries**

Netherlands

**Chiara de Waure**

Italy

**John de Wit**

Australia

**Kora DeBeck**

Canada

**Scott Decker**

USA

**Kathleen Deering**

Canada

**Margaret (Greta) Defeyter**

UK

**Benedicte Deforche**

Belgium

**Olivier Degomme**

Belgium

**Kianoush Dehghani**

Canada

**Francine Dehue**

Netherlands

**Marloes Dekker Nitert**

Australia

**Rosa del Angel**

Mexico

**Felicitas Dela Cruz**

USA

**Paul Delamarche**

France

**Toni Delaney**

Australia

**Juan Delgado**

Guatemala

**Tom Deliens**

Belgium

**Hélène Delisle**

Canada

**Andrea DeLuca**

USA

**Carol DeMatteo**

Canada

**Evangelia Demou**

UK

**JM (Lea) den Broeder**

Netherlands

**Casper den Heijer**

Netherlands

**Gerald V. Denis**

USA

**Tekalign Deressa**

Ethiopia

**Kebede Deribe**

Ethiopia

**Amare Deribew**

Ethiopia

**Ilse Derluyn**

Belgium

**Richard Derman**

USA

**Wayne deRuiter**

Canada

**Jean-Claude Desenclos**

France

**Sameer Deshpande**

Canada

**Ann DeSmet**

Belgium

**Walter Devillé**

Netherlands

**Amanda Devine**

Australia

**Subhojit Dey**

India

**Nepal Dey**

Bangladesh

**Nafeesa Dhalwani**

UK

**Mukesh Dherani**

UK

**Meghnath Dhimal**

Nepal

**Aldo Di Carlo**

Italy

**Sonia Dias**

Portugal

**Esperanza Diaz**

Norway

**Alejandro Diaz**

Argentina

**María del Pilar Díaz**

Argentina

**Brenda Diergaarde**

USA

**Ana Diez-Fernandez**

Spain

**Maartje Dijkstra**

Netherlands

**Jo-Anne R Dillon**

Canada

**Charles DiMaggio**

USA

**Joanna Dipnall**

Australia

**Artur Direito**

New Zealand

**Joseph Ditre**

USA

**Nicola Diviani**

Switzerland

**Helen Dixon**

Australia

**Mai Do**

USA

**Ha Do**

Vietnam

**Naa Dodua Dodoo**

Ghana

**Irene Doherty**

USA

**David Teye Doku**

Ghana

**James Dollman**

Australia

**Ana Luiza Domingos**

Brazil

**Antonia Domingo-Salvany**

Spain

**Benjamin Domingue**

USA

**M Felícitas Domínguez-Berjón**

Spain

**Carolin Donath**

Germany

**Amol Dongre**

India

**Paul Donnelly**

UK

**Tam Donnelly**

Canada

**Philippe Donnen**

Belgium

**Lucy Doos**

UK

**Aimee Dordevic**

Australia

**Maria Dorrucci**

Italy

**Svetlana Vladislavovna Doubova**

Mexico

**Margaret Douglas**

UK

**Mary Dowd**

USA

**Julia Downing**

Uganda

**Katherine Downing**

Australia

**Shauna Downs**

USA

**Christopher Dowrick**

UK

**Ann Dozier**

USA

**Bettina Drake**

USA

**Alison Drake**

USA

**Catherine Draper**

South Africa

**Jessica Draughon**

USA

**Jessica Draughon Moret**

USA

**Jonathan Drennan**

UK

**Clemens Drenowatz**

USA

**Clemens Drenowatz**

USA

**Hans Drexler**

Germany

**Constance Drossaert**

Netherlands

**Nicolas Droste**

Australia

**Jean-Baptist du Prel**

Germany

**Eve Dubé**

Canada

**David DuBois**

USA

**Helena Duch**

USA

**Pat Dudgeon**

Australia

**Dean Dudley**

Australia

**Martin Duerden**

UK

**Marylene Dugas**

Canada

**Pauline Duke**

Canada

**Ian Duncan**

USA

**Mitch J Duncan**

Australia

**Matthew Dunn**

Australia

**Sheila Dunn**

Canada

**James Dunn**

Canada

**Michael Dunne**

Australia

**John Duperly**

Colombia

**Jo Durham**

Australia

**Gareth Dutton**

USA

**Genevieve Dwyer**

Australia

**Laura Dwyer-Lindgren**

USA

**Anne Margarita Dyrhol-Riise**

Norway

**Pam Dyson**

UK

**Matthew Eastin**

USA

**Keith Eastwood**

Australia

**Bassey Ebenso**

UK

**Ericka Ebot**

USA

**Kristin Eccles**

USA

**Kate Eddens**

USA

**David Ederer**

USA

**Kristina Edvardsson**

Sweden

**Charlotte Edwardson**

UK

**Frida Eek**

Sweden

**Valery Effoe**

USA

**Sander Matthijs Eggers**

Netherlands

**Carolyn Ehrlich**

Australia

**Annahita Ehsan**

Canada

**Randi Eikeland**

Norway

**Rochelle Eime**

Australia

**Manuel Eisner**

UK

**Muslima Ejaz**

Pakistan

**Solvig Ekblad**

Sweden

**Christopher Bismarck Eke**

Nigeria

**Chris Ekpenyong**

Nigeria

**Eva-Charlotte Ekström**

Sweden

**Anette Ekström**

Sweden

**Osa-Eloka Ekwueme**

Nigeria

**Obinna Ikechukwu Ekwunife**

Germany

**Noura El Salibi**

Lebanon

**Rajavel Elango**

Canada

**M El-Ashker**

USA

**Jessica Elf**

USA

**Abdel-Hady El-Gilany**

Egypt

**Bent-Martin Eliassen**

Norway

**Leticia Elizondo-Montemayor**

Mexico

**Michael Elliott**

USA

**Susan Elliott**

Canada

**Matthew Ellis**

UK

**Philippa Ellwood**

New Zealand

**Khalifa Elmusharaf**

Sudan

**Maged El-Setouhy**

Saudi Arabia

**Gretchen Ely**

USA

**Joanne Embree**

Canada

**Ulrika Enemark**

Denmark

**Lina Engelen**

Australia

**Paul Enright**

USA

**Malin Eriksson**

Sweden

**Tor Eriksson**

Denmark

**Thérèse Eriksson**

Sweden

**Ksenia Eritsyan**

Russian Federation

**Berhanu Erko**

Ethiopia

**Andrea Ermolao**

Italy

**Linda Ernstsen**

Norway

**Sebhat Erqou**

USA

**Steven Ersser**

UK

**Jenni Ervasti**

Finland

**Karl Eschbach**

USA

**Jorge Escobedo**

Mexico

**Cam Escoffery**

USA

**Beverley Essue**

Canada

**Ekpereonne Esu**

Nigeria

**Adrienne Ettinger**

USA

**Perez Eusebio**

Mexico

**Catrin Evans**

UK

**Charlotte Evans**

UK

**Subhadra Evans**

USA

**Rebecca Evans**

Australia

**Barbara Evans**

United Arab Emirates

**Charlotte Evans**

UK

**Alexandra Evans**

USA

**Ben Ewald**

Australia

**Daniel Exeter**

New Zealand

**Emma Eyre**

UK

**Eugene Ezebilo**

Sweden

**Mangi Ezekiel**

Tanzania

**Nicole Ezendam**

Netherlands

**Pam Factor-Litvak**

USA

**Jessica Fales**

USA

**Debbie Fallon**

UK

**Fan Fan**

China

**Hildegunn Fandrem**

Norway

**Jiming Fang**

Canada

**Jessica Fanzo**

USA

**Christopher Farah**

USA

**Daniel Farkas**

USA

**Ahmed Fathelrahman**

Saudi Arabia

**Akinola Fatiregun**

Nigeria

**Zafar Fatmi**

Pakistan

**Lanfranco Fattorini**

Italy

**Alex Faulkner**

UK

**Rebecca Fauth**

USA

**Bukola Fawole**

Nigeria

**Olufunmilayo Fawole**

Nigeria

**Bukola Fawole**

Nigeria

**Mina Fazel**

UK

**Denise Fearman Johnson**

USA

**Ugo Fedeli**

Italy

**Bruno Federico**

Italy

**Frank Feeley**

USA

**Berhanu Feleke**

Ethiopia

**Eleonora Feletto**

Australia

**Xiaoqi Feng**

Australia

**Nora Fenske**

Germany

**Ruwan Ferdinando**

Sri Lanka

**Jill Ferdinands**

USA

**Laura Ferguson**

USA

**Christopher Ferguson**

USA

**Jose Fernandez**

USA

**Juan Miguel Fernandez-Alvira**

Spain

**Sumadhya Fernando**

Sri Lanka

**Katia Ferrar**

Australia

**Jason Ferris**

Australia

**Leopold K Fezeu**

France

**Marie Fialkowski**

USA

**Maria Fiatarone Singh**

Australia

**Thiago Fidalgo**

Brazil

**Robert Field**

USA

**Rebecca Fielding-Miller**

USA

**David Fields**

USA

**Albert Figueras**

Spain

**Bianca Fileborn**

Australia

**Antonietta Filia**

Italy

**Filippos Filippidis**

UK

**Aurélie Fillion**

France

**Suzanne Filteau**

UK

**Giulia Fioravanti**

Italy

**Katherine Fiori**

USA

**Colin Fischbacher**

UK

**Frida Fischer**

Brazil

**Joan Fischer**

USA

**Niamh Fitzgerald**

UK

**Eugene Fitzhugh**

USA

**Ewa Florek**

Poland

**Alina Flores**

USA

**Maria Cristina Flores Soares**

Brazil

**Albert Flynn**

Ireland

**Joseph Fokam**

Cameroon

**Morenike Folayan**

Nigeria

**Sara Folta**

USA

**Virginia Fonner**

USA

**Maria João Fonseca**

Portugal

**Cynthia Forbes**

Canada

**Dara Ford**

USA

**Peta Forder**

Australia

**Catherine Forestell**

USA

**Maria Joao Forjaz**

Spain

**Lynne Forrest**

UK

**Alice Forster**

UK

**Elisabet Forsum**

Sweden

**Ann Forsyth**

USA

**Claire Foster**

UK

**Lyndie Foster Page**

New Zealand

**Heather Fowler**

USA

**Joel Francis**

UK

**Molly Franke**

USA

**Richard Franklin**

Australia

**Eleonor Fransson**

Sweden

**Jose Frantz**

South Africa

**Rosanne Freak-Poli**

Australia

**John Frean**

South Africa

**Yoni Freedhoff**

Canada

**Michael Freeman**

USA

**Rebecca French**

UK

**Clare French**

UK

**Leah Frerichs**

USA

**Nicholas Freudenberg**

USA

**Paula Frew**

USA

**Christine Friedenreich**

Canada

**Elliot Friedman**

USA

**Rebecca Fry**

USA

**Robert Fryatt**

South Africa

**Robert Fryatt**

South Africa

**Victoria Frye**

USA

**John Fu**

USA

**Yongshui Fu**

China

**Hongyun Fu**

USA

**Liza Fuentes**

USA

**Kimberly Fulda**

USA

**Pauline Fuller**

UK

**Matthew Fuller-Tyszkiewicz**

Australia

**Laura Fumanelli**

Italy

**Martha Funnell**

USA

**Adam Fusheini**

South Africa

**Maria Fuster**

Spain

**Li Fuzhong**

USA

**Giovanni Gabutti**

Italy

**Thierry Gagné**

Canada

**France Gagnon**

Canada

**Daniel Galanis**

USA

**Seana Gall**

Australia

**Robyn Gallagher**

Australia

**Katherine Gallagher**

UK

**Danielle Gallegos**

Australia

**Katia Gallegos-Carrillo**

Mexico

**Silvano Gallus**

Italy

**Tais Galvão**

Brazil

**Sofia Gameiro**

UK

**Rashmi Gangamma**

USA

**Matthew Ganio**

USA

**Hayley Gans**

USA

**Marie Gantz**

USA

**Mette Gantzhorn**

Denmark

**Yang Gao**

Hong Kong

**James Garbutt**

USA

**Maria Garcia-Gil**

Spain

**Anne Helene Garde**

Denmark

**Benjamin Gardner**

UK

**Lauren Gardner**

Australia

**Pernilla Garmy**

Sweden

**Anastasia Garoufi**

Greece

**Mario D. Garrett**

USA

**Jennifer Garvin**

USA

**Danijela Gasevic**

UK

**Gasim Gasim**

Sudan

**Tania Gaspar**

Portugal

**Rui Gaspar**

Portugal

**Roberto Gasparini**

Italy

**Peter Gates**

Australia

**Derek Gatherer**

UK

**Emma Gearon**

Australia

**Klaus Gebel**

Australia

**Rosemary Geddes**

UK

**Molla Gedefaw**

Ethiopia

**Sarah Gee**

New Zealand

**Sarah Geiger**

USA

**Nancy Gell**

USA

**Becky Genberg**

USA

**Usha George**

Canada

**Christine George**

USA

**Sanne Gerards**

Netherlands

**Dorota Gertig**

Australia

**Hailay Gesesew**

Ethiopia

**Dionne Gesink**

Canada

**Marjolein Geurts**

Netherlands

**Ata Ghaderi**

Sweden

**Akhgar Ghassabian**

USA

**Musie Ghebremichael**

USA

**Silvia Ghirini**

Italy

**Subharati Ghosh**

India

**Minelli Giada**

Italy

**Sigrid Gibson**

UK

**Melanie Gibson-Helm**

Australia

**Jasmine Gideon**

UK

**Victoria Gilbart**

UK

**Diana Gil-González**

Spain

**Christopher Gill**

USA

**Simone Gill**

USA

**Ardyth Gillespie**

USA

**Conor Gilligan**

Australia

**Richard Gillum**

USA

**Suely Godoy Agostinho Gimeno**

Brazil

**Rosa Gini**

Italy

**Tamar Ginossar**

USA

**Gary Ginsberg**

Israel

**Paolo Giorgi Rossi**

Italy

**Smith Giri**

Nepal

**Belaineh Girma**

Ethiopia

**Eshetu Girma**

Ethiopia

**Samuel Girma**

Ethiopia

**Robert Gish**

USA

**LisaAnn Gittner**

USA

**Massimo Giuliani**

Italy

**Marina Giuliano**

Italy

**Stanton Glantz**

USA

**Sabine Glock**

Germany

**Mark Glover**

UK

**Anne Glowinski**

USA

**Nick Glozier**

Australia

**Wanzahun Godana**

Ethiopia

**Jacques Godfroid**

Norway

**Elisabeth Goffeng**

Norway

**Julia Gohlke**

USA

**Jeanne Goldberg**

USA

**Sharon Goldfeld**

Australia

**Sidra Goldman-Mellor**

USA

**Edward Goldstein**

USA

**Devon Golem**

USA

**Yvonne M. Golightly**

USA

**Jonathan Golub**

USA

**Alex Gomez Bruton**

Spain

**Rita A. Gomez-Diaz**

Mexico

**Denise Utsch Gonçalves**

Brazil

**Cathy Gong**

Australia

**Raquel Gonzalez**

Spain

**Melody Goodman**

USA

**Louisa Gordon**

Australia

**Giuseppe Gorini**

Italy

**Kathleen Gorman**

USA

**Yevgeniy Goryakin**

UK

**James Gosney**

USA

**Neela Goswami**

USA

**Atsushi Goto**

Japan

**Dinah Gould**

USA

**Annabelle Gourlay**

UK

**Mary Grabowski**

USA

**Mark Grabowsky**

USA

**Kathryn Grace**

USA

**Luis Gracia-Marco**

UK

**Guendalina Graffigna**

Italy

**Jay Graham**

USA

**Andrew Grandinetti**

USA

**Michael Grandner**

USA

**Genevieve Grant**

Australia

**Marcus Grant**

UK

**Alberto Grao Cruces**

Spain

**Janessa Graves**

USA

**Stuart Gray**

UK

**Cindy Gray**

UK

**Matt Graziose**

USA

**Ezekiel Green**

South Africa

**Jennifer Greif Green**

USA

**Andrew Greenhill**

Australia

**Jeffrey Greeson**

USA

**Jessica Grieger**

Australia

**Rosane Griep**

Brazil

**Larissa Grigoryan**

USA

**Eva Grill**

Germany

**Maria Grillet**

Venezuela

**Ulrike Grittner**

Germany

**Pam Groenewald**

South Africa

**Angelique Grosser**

Germany

**Heiner Grosskurth**

UK

**Ashley Grosso**

USA

**Franziska Grossschaedl**

Austria

**Johannes Gruber**

Germany

**Oliver Gruebner**

USA

**John Grundy**

Australia

**Anne Grunseit**

Australia

**Raphael Grzebieta**

Australia

**Maciej Grzybek**

Poland

**Justin Guagliano**

USA

**Emanuela Gualdi**

Italy

**Jessica Sophia Gubbels**

Netherlands

**Kimberly Gudzune**

USA

**Paulo Guerra**

Brazil

**Gilles Guerrier**

France

**Montserrat Guillen**

Spain

**Patricia Guimaraes**

Brazil

**Hayley Guiney**

New Zealand

**Benjamin Guinhouya**

France

**Anil Gumber**

UK

**Freedom Nkhululeko Gumedze**

South Africa

**Nalika Gunawardena**

Sri Lanka

**Daniel Gundersen**

USA

**RB Gunier**

USA

**Janelle Gunn**

USA

**Fei-Ran Guo**

Taiwan

**Jong-Long Guo**

Taiwan

**Yan Guo**

China

**Jing Guo**

China

**Hongxiong Guo**

China

**Jong-Long Guo**

Taiwan

**Neeru Gupta**

India

**Vidhu Gupta**

India

**Micheal Gurven**

USA

**Narcis Gusi**

Spain

**Juan Luis Gutierrez-Fisac**

Spain

**David Guwatudde**

Uganda

**Giorgio Guzzetta**

Italy

**Clement Gwede**

USA

**Zsuzsa Gyorffy**

Hungary

**Ranjani H**

India

**David Haaga**

USA

**Steven Haas**

USA

**Gerda-Maria Haas**

Germany

**Muhammad Habib**

Canada

**Rima Habib**

Lebanon

**Erin Hager**

USA

**Ali Akbar Haghdoost**

Iran

**Nao Hagiwara**

USA

**Hyeouk Chris Hahm**

USA

**Jess Haines**

Canada

**Anjum Hajat**

USA

**Nemat Hajeebhoy**

Vietnam

**Magnus Hakeberg**

Germany

**Lauren A. Haldeman**

USA

**Amal Krishna Halder**

Bangladesh

**Arja Halkoaho**

Finland

**Casey Hall**

USA

**Mats Hallgren**

Sweden

**Jaana Halonen**

Finland

**Bonnie Halpern-Felsher**

USA

**Randah Hamadeh**

Bahrain

**Gabriel Hamer**

USA

**Jan Hamling**

UK

**Anne Hammarström**

Sweden

**Regina Hampel**

Germany

**Bing Han**

USA

**Jill Hanass-Hancock**

South Africa

**Andria Hanbury**

UK

**Nancy Hancock**

USA

**Tonelle Handley**

Australia

**Sebastien Haneuse**

USA

**Catherine Hankey**

UK

**Charlotte Hanlon**

Ethiopia

**Liz Hanna**

Australia

**Åse Marie Hansen**

Denmark

**Claus D. Hansen**

Denmark

**Kjell Hansson Mild**

Sweden

**Given Hapunda**

Zambia

**Farhana Haque**

Bangladesh

**Md. Aminul Haque**

Bangladesh

**Jessica Harding**

Australia

**Louise Hardy**

Australia

**Guy Harling**

USA

**Shamil Haroon**

UK

**Jane Harries**

South Africa

**Patrick Harris**

Australia

**Fiona Harris**

UK

**Abigail Harrison**

USA

**Rosane Härter Griep**

Sweden

**Eileen Harwood**

USA

**Rumina Hasan**

Pakistan

**Penelope Hasking**

Australia

**David Haslam**

UK

**Theresa Hastert**

USA

**Abigail Hatcher**

South Africa

**Angelos Hatzakis**

Greece

**Sally Haw**

UK

**Marquis Hawkins**

USA

**Nicola Hawley**

USA

**Kanna Hayashi**

Canada

**Mary Hayden**

USA

**Melanie Hayman**

Australia

**Ann Haynos**

USA

**Af Haynos**

USA

**Na He**

China

**Yanling He**

China

**Debra Hector**

Australia

**Craig Hedberg**

USA

**Kristiann Heesch**

Australia

**Kelsey Hegarty**

Australia

**Banafsheh Heidari**

Iran

**Motahar Heidari-Beni**

Iran

**Robert Heimer**

USA

**Asgeir R. Helgason**

Sweden

**Stephane Helleringer**

USA

**Shaun Helman**

UK

**Sheryl Hemphill**

Australia

**Claire Henderson**

UK

**Eilis Hennessy**

Ireland

**E.A Hennessy**

USA

**Bonnie Henry**

Canada

**Stanley Henshaw**

USA

**Ebiere Herbertson**

Nigeria

**Florian Herbolsheimer**

Germany

**Jane Herlihy**

UK

**Joumana Hermez**

Egypt

**Sonia Hernandez-Cordero**

Mexico

**Victoria Hernando**

Spain

**Manuel Herrador-Colmenero**

Spain

**Oscar Fernando Herran Falla**

Colombia

**Sharon Herring**

USA

**Steven Herrmann**

USA

**Kathryn Hesketh**

UK

**Michelle Hesse**

USA

**Ingrid Heuch**

Norway

**Margaret Hicken**

USA

**Natalia Hidalgo-Ruzzante**

Spain

**Heikki Hiilamo**

Finland

**Alison Hill**

Australia

**Ryan Hill**

USA

**Frances Hillier-Brown**

UK

**Danny Hills**

Australia

**Erica Hinckson**

New Zealand

**Sven Gudmund Hinderaker**

Norway

**Melanie Hingle**

USA

**Trina Hinkley**

Australia

**Antoine Vikkey Hinson**

Benin

**Taina Hintsa**

Finland

**David Hipgrave**

Australia

**James Hipp**

USA

**Yoichiro Hirakawa**

Australia

**Yoichiro Hirakawa**

Australia

**Caroline Hird**

UK

**Jennifer Hirsch**

USA

**Rosemary Hiscock**

UK

**Ayako Hiyoshi**

Sweden

**Heidi Hjelmeland**

Norway

**Jill Hnatiuk**

Australia

**Mandy Ho**

Australia

**Emma Hock**

UK

**Ian Hodgson**

UK

**Christian Hoebe**

Netherlands

**Nancy Hoeymans**

Netherlands

**Robert Hoffman**

USA

**Daniel Hoffman**

USA

**Heather Hoffman**

USA

**Sandra Hoffmann**

USA

**Falk Hoffmann**

Germany

**Kathryn Hoffmann**

Austria

**Lesley Hoggart**

UK

**Theresa Hoke**

USA

**Clare Holdsworth**

UK

**Michael Holick**

USA

**Anna-Clara Hollander**

Sweden

**Katie Holliday**

USA

**Daniel Holman**

UK

**King Holmes**

USA

**Bev J Holmes**

Canada

**Bjørn E. Holstein**

Denmark

**Martin Holt**

Australia

**Knut Holtedahl**

Norway

**Lori Holtz**

USA

**David Hondula**

USA

**Jan Hontelez**

Netherlands

**Claire Hooker**

Australia

**Steven Hooker**

USA

**Lan T. Ho-Pham**

Vietnam

**Monjurul Hoque**

South Africa

**Tanya Horacek**

USA

**Willi Horner-Johnson**

USA

**Jennifer Horney**

USA

**Susan Horton**

Canada

**H Dean Hosgood**

USA

**Jamie Hosking**

New Zealand

**Nazli Hossain**

Pakistan

**Golam Hossain**

Bangladesh

**Christine Hotz**

Canada

**Jonathan Houdmont**

UK

**Inge Houkes**

Netherlands

**James House**

USA

**Brian Houston**

USA

**Hanno Hoven**

Germany

**Ciska Hoving**

Netherlands

**Natasha Howard**

Australia

**Annie Green Howard**

USA

**Nopporn Howteerakul**

Thailand

**Kelly Hsieh**

USA

**Howard Hu**

USA

**Shu Hu**

Singapore

**Yaoyue Hu**

UK

**Guoqing Hu**

China

**Yi Hu**

China

**Gang Hu**

USA

**Chiung-Yu Huang**

Taiwan

**Haiou Huang**

USA

**Jennifer Huberty**

USA

**Amanda Hughes**

USA

**John Hughes**

UK

**Jimi Huh**

USA

**Andrew Hull**

USA

**Pamela Hull**

USA

**Chifa Hung**

Taiwan

**Kate Hunter**

Australia

**Susie Huntington**

UK

**John Hurdle**

USA

**Rafat Hussain**

Australia

**Azmina Hussain**

Pakistan

**Mehwish Hussain**

Pakistan

**Jasmin Hutchinson**

USA

**Dyfed Huws**

UK

**Ronald Iannotti**

USA

**Jennifer Ibrahim**

USA

**Joseph Ibrahim**

Australia

**Scott Ickes**

USA

**Ellen Idler**

USA

**Zubairu Iliyasu**

Nigeria

**Samantha Illangasekare**

USA

**Roger Ingham**

UK

**Loredana Ingrosso**

Italy

**Okafor Innocent Igwebueze**

Nigeria

**Kaire Innos**

Estonia

**Tabassum Insaf**

USA

**Brandon Irwin**

USA

**Jennifer D. Irwin**

Canada

**Anton Isaacs**

Australia

**Sheila Isanaka**

France

**Yoshiki Ishikawa**

Japan

**Tatsuro Ishizaki**

Japan

**Talat Islam**

USA

**Talat Islam**

USA

**Shariful Islam**

Germany

**Md Rafiqul Islam**

Australia

**Lindsey Jaacks**

USA

**Kauser Jabeen**

Pakistan

**Michael Jackson**

USA

**Kristina Jackson**

USA

**Julia Jaekel**

USA

**Kirstie Jagoe**

Canada

**Sachin Jain**

USA

**Camilla Jalling**

Sweden

**Mohamed Jalloh**

Senegal

**Syed Tanzil Jamali**

Pakistan

**Tanzil Jamali**

Pakistan

**Bryan James**

USA

**Carole James**

Australia

**Muhammad Jamil**

Australia

**Jonine Jancey**

Australia

**Monika Janda**

Australia

**Elena Jansen**

Australia

**Pauline Jansen**

Nehterlands

**Petrus Jansen van Vuren**

South Africa

**Staffan Janson**

Sweden

**Fanny Janssen**

Netherlands

**Heidi Janssens**

Belgium

**Pande Putu Januraga**

Indonesia

**Megan Jarman**

UK

**Patricia Jarosz**

USA

**Jordan Jarvis**

USA

**Barbara Jauregui**

USA

**Marjan Javanbakht**

USA

**Ahsin Jawad**

UK

**Rama Jayaraj**

Australia

**Caremel Jean-Francois**

Niger

**Rajesh Jeewon**

Mauritius

**Maria Elena Jefferds**

USA

**Barbara Jefferis**

UK

**Kevin Jefferson**

USA

**Alison Jeffery**

UK

**Robert W. Jeffery**

USA

**Frederic Jehan**

France

**Marion W Jenkins**

USA

**Nancy A. Jennings**

USA

**Chris Jensen**

Denmark

**Jorgen Dejgaard Jensen**

Denmark

**Degu Jerene**

Ethiopia

**David Jernigan**

USA

**Marc Jeuland**

USA

**Rachel Jewkes**

South Africa

**Bu-Tian Ji**

USA

**Cun-Xian Jia**

China

**Yingnan Jia**

China

**Masamine Jimba**

Japan

**Andrew Jin**

Canada

**Fengyi Jin**

Australia

**Tian Jingyan**

China

**Mark Jit**

UK

**Booil Jo**

USA

**Matti Joensuu**

Finland

**Junia Joffer**

Sweden

**Michel Joffres**

Canada

**Rijo John**

India

**Helle Laier Johnsen**

Norway

**Roar Johnsen**

Norway

**Timothy Johnson**

USA

**Tammie Johnson**

USA

**Janice Johnston**

Hong Kong

**Kate Jolly**

UK

**Anna Jones**

UK

**Brenda Jones**

USA

**Tiffany Jones**

Australia

**Cheryl Jones**

Australia

**Andy Jones**

UK

**Jessica Jones-Smith**

USA

**Robert Jonzon**

Sweden

**Rashid Jooma**

Pakistan

**Melanie Jordan**

UK

**Brigid Jordan**

Australia

**María José González-Muñoz**

Spain

**Rodney Joseph**

USA

**Hari Shanker Joshi**

India

**Rohina Joshi**

Australia

**Shashank Joshi**

India

**Roshni Joshi**

UK

**Hari Shanker Joshi**

India

**Grace Joshy**

Australia

**Aleksandra Jovic Vranes**

Serbia

**Paul Juarez**

USA

**Sol Juárez**

Sweden

**Kyoungrae Jung**

USA

**Eunok Jung**

South Korea

**Annemarie Jutel**

New Zealand

**Zubair Kabir**

Ireland

**Humayun Kabir**

Bangladesh

**Zubair Kabir**

Ireland

**Maria Kaczmarek**

Poland

**Andrew. T Kaczynski**

USA

**Gibson Benard Kagaruki**

Tanzania

**Masaharu Kagawa**

Japan

**Benjamin Kagina**

South Africa

**Mayumi Kako**

Australia

**John Kalbfleisch**

USA

**Pauline Kale**

Brazil

**Dorota Kaleta**

Poland

**Dorota Kaleta**

Poland

**Håkan Källmén**

Sweden

**Ummi Kalsum**

Indonesia

**Debra Kaminer**

South Africa

**Kelly Kamm**

USA

**Dorcas Kamuya**

Kenya

**Bavesh Davandra Kana**

South Africa

**Musa Kana**

Nigeria

**Namratha Kandula**

USA

**Hyun-Sik Kang**

South Korea

**Almamy Kanté**

Tanzania

**Steve Kanters**

Canada

**Suman Kanungo**

India

**Syeda Kanwal**

Pakistan

**Rachel Kaplan**

USA

**Satwanti Kapoor**

India

**Amalia Karahalios**

Australia

**E Karam**

Lebanon

**Sharvari Karandikar**

USA

**Maria Karanika-Murray**

UK

**Panagiotis Karanis**

China

**Rajendra Karkee**

Nepal

**Carrie Karvonen-Gutierrez**

USA

**Rosemin Kassam**

Canada

**Christina-Maria Kastorini**

Greece

**Tarun Reddy Katapally**

Canada

**David Katende**

Uganda

**Kendra Kattelmann**

USA

**Joanne Katz**

USA

**David Katz**

USA

**Rebecca Katz**

USA

**Michelle Kaufman**

USA

**B Kauhl**

Netherlands

**Deepak Kaul**

India

**Ravneet Kaur**

India

**Cemil Kavalci**

Turkey

**Anne Kavanagh**

Australia

**Rob Kavet**

USA

**SM Kayaga**

UK

**Bengt Kayser**

Switzerland

**Lawrence Kazembe**

Namibia

**Sarah Keadle**

USA

**Christopher Kearney**

USA

**Benjamin Kearns**

UK

**Daniel Keating**

USA

**Getahun Kebede Beyera**

Ethiopia

**Aleksandar Kecojevic**

USA

**Richard Keegan**

Australia

**Tessa Keegel**

Australia

**Helen Keeley**

Ireland

**Mpho Keetile**

Botswana

**Shannon Kehle-Forbes**

USA

**Alexander Keil**

USA

**Linda Kelemen**

Canada

**Cecily Kelleher**

Ireland

**Scott Kellerman**

USA

**Adrian Kelly**

Australia

**Paul Kelly**

UK

**Matthew Kelly**

Australia

**Louise Kelly**

USA

**Angela Kelly-Hanku**

Papua New Guinea

**Louise Kelly-Hope**

UK

**Han Cg Kemper**

Netherlands

**Eben Kenah**

USA

**Garth Kendall**

Australia

**Nancy Kendall**

USA

**Garth Kendall**

Australia

**Friederike Kendel**

Germany

**Hal Kendig**

Australia

**Mustafa Kendirci**

Turkey

**Andre Pascal Kengne**

South Africa

**Gina Kennedy**

Italy

**Norelee Kennedy**

Ireland

**Catriona Kennedy**

Ireland

**Amanda Kenny**

Australia

**Ernest Kenu**

Ghana

**Peter Keogh**

UK

**Marko Kerac**

UK

**Bernard Keraita**

Ghana

**Jessica Keralis**

USA

**Brent Kerger**

USA

**Jocelyn Kernot**

Australia

**Jean Kerver**

USA

**Ronald Kessler**

USA

**Joanna Kesten**

UK

**Yan Kestens**

Canada

**K Ketema**

USA

**Oili Kettunen**

Finland

**Zarnie Khadjesari**

UK

**MMH Khan**

Germany

**Yasmin Khan**

Canada

**Hasinur Rahaman Khan**

Bangladesh

**Farida Habib Khan**

Saudi Arabia

**Tahir Khan**

Pakistan

**Salah Uddin Khan**

USA

**Vishnu Khanal**

Nepal

**Ashrafunnesa Khanom**

UK

**Marwan Khawaja**

Lebanon

**Davoud Khorasani-Zavareh**

Sweden

**Z Khoury**

Brazil

**Simon Peter Kibira**

Uganda

**Solomon Kibret**

Ethiopia

**Sean Kidd**

Canada

**Judi Kidger**

UK

**Rachel Kidman**

USA

**Michaela Kiernan**

USA

**Dorota Kiewra**

Poland

**Manay Kifle**

Ethiopia

**Reinhold Kilian**

Germany

**Daejung Kim**

South Korea

**Juhee Kim**

USA

**Susan Kim**

Australia

**Sun-Young Kim**

South Korea

**Brian Kim**

USA

**Ruth Kimokoti**

USA

**Andy King**

USA

**Romaine King**

USA

**Melanie Kingsland**

Australia

**Janni Kinsler**

USA

**Susan Kippax**

Australia

**Ruth Kipping**

UK

**Anette Kira**

New Zealand

**Sharon Kirkpatrick**

Canada

**Irma Kirtadze**

Georgia

**Philippe Kiss**

Belgium

**Cari Kitahara**

USA

**Scott Kitchener**

Australia

**JM Kittner**

Germany

**Evan Kleiman**

USA

**Terry Klein**

USA

**Knut-Inge Klepp**

Norway

**Kerstin Klipstein-Grobusch**

Netherlands

**A Kloch**

Poland

**Jens Klotsche**

Germany

**Antti Knaapila**

Finland

**Thomas Kneib**

Germany

**Lee Knifton**

UK

**Stephen Knight**

South Africa

**Keegan Knittle**

Finland

**Cody Knutson**

USA

**Lindsay Kobayashi**

UK

**Pamela Koch**

USA

**Linda Koenig**

USA

**Mallory Koenings**

USA

**Stefan Kohler**

Germany

**Pamela Kohler**

USA

**Gerjo Kok**

Netherlands

**Lauri Kokkinen**

Finland

**Tracy Kolbe-Alexander**

Australia

**Keiko Kondo**

Japan

**Naoki Kondo**

Japan

**Grace Kong**

USA

**Pornpimol Kongtip**

Thailand

**Evangelos Kontopantelis**

UK

**Wendy Koolhaas**

Netherlands

**Liisa Korkalo**

Finland

**Siran Koroukian**

USA

**Mette Korshøj**

Denmark

**Jesse Kosiba**

USA

**Kristine Koski**

Canada

**Michael Kostapanos**

Greece

**Deliana Kostova**

USA

**Emily Kothe**

Australia

**Christiana Kouta**

Cyprus

**Anne Kouvonen**

Finland

**Fiona Kouyoumdjian**

Canada

**John Koval**

Canada

**Viviane Kovess**

France

**Bernd Kowall**

Germany

**Augustine Kposowa**

USA

**Alexander Kraemer**

Germany

**Nir Krakauer**

USA

**Douglas Krakower**

USA

**Gunilla Krantz**

Sweden

**Allan Krasnik**

Denmark

**Gary Krieger**

USA

**Kristian Krieger**

Belgium

**Dean Kriellaars**

Canada

**Triveni Krishnan**

India

**GV Krishnaveni**

India

**Petter Kristensen**

Norway

**Jesper Kristiansen**

Denmark

**Alfgeir Logi Kristjansson**

USA

**Vicki Kristman**

Canada

**Mareike Kroll**

Germany

**Grace Marie Ku**

Philippines

**Renata Kuciene**

Lithuania

**Alexis Kuerbis**

USA

**Mirte Kuipers**

Netherlands

**Ralf Kuja-Halkola**

Sweden

**Jennifer Kuk**

Canada

**Pamela Kulbok**

USA

**Abhishek Kumar**

Germany

**G Anil Kumar**

India

**Subodh Kumar**

India

**Ramya Kumar**

Sri Lanka

**Jharna Kumbang**

UK

**Anton Kunst**

Netherlands

**Sofie Kuppens**

Belgium

**Stan Kutcher**

Canada

**Keisuke Kuwahara**

Japan

**Dominika Kwasnicka**

Australia

**Jesse Kwiek**

USA

**Japheth Kwiringira**

Uganda

**Elisabeth Kylberg**

Sweden

**Theodore Kypraios**

UK

**Amos Laar**

Ghana

**Edmar Lacerda**

Brazil

**Dirk Lachenmeier**

Germany

**Francine Laden**

USA

**Joel Ladner**

France

**Sophie Laforest**

Canada

**Maria João Lagoa**

Portugal

**Nathalie Lahoud**

Lebanon

**Wen-Ter Lai**

Taiwan

**Yihunie L Lakew**

Ethiopia

**Cynthia Lakon**

USA

**Tina Lam**

Australia

**Hugh Simon Hung San Lam**

Hong Kong

**Kin Bong Hubert Lam**

UK

**Molly Lamb**

USA

**Karen Lamb**

Australia

**Poppy Lamberton**

UK

**Yvonne Lamers**

Canada

**Joel Lamounier**

Brazil

**S Lamy**

France

**Dadja Essoya Landoh**

Togo

**Britta Lang**

United Arab Emirates

**John Lange**

USA

**Kylie Lange**

Australia

**Rebecca Langford**

UK

**Tessa Langley**

UK

**Kamran Lankarani**

Iran

**Amy Joy Lanou**

USA

**Francesca Lantieri**

Italy

**Xiang Qian Lao**

Hong Kong

**Malinee Laopaiboon**

Thailand

**Richard Larouche**

Canada

**Amparo Larrauri**

Spain

**Charles Larson**

Canada

**Bruce Larson**

USA

**Heidi Larson**

UK

**Kayla Laserson**

India

**Laura Laslett**

Australia

**Mahbub Latif**

Bangladesh

**Robab Latifnejad Roudsari**

Iran

**Janet Latner**

USA

**Patrick Wing Chung Lau**

Hong Kong

**Alexis Lauricella**

USA

**Paolo Lauriola**

Italy

**Gunter Laux**

Germany

**Anthony Laverty**

UK

**Angela Lawless**

Australia

**Sharon Lawn**

Australia

**Saranath Lawpoolsri**

Thailand

**EM Lawrence**

USA

**M. Barton Laws**

USA

**Hal Lawson**

USA

**Beverley Lawton**

New Zealand

**Lisa Lazarus**

Canada

**DeAnn Lazovich**

USA

**Lambros Lazuras**

Greece

**Giacomo Lazzeri**

Italy

**Marzia Lazzerini**

Italy

**Tricia Leahey**

USA

**Scott Leatherdale**

Canada

**Justine Leavy**

Australia

**James LeCheminant**

USA

**Chris Leck**

UK

**Annette Leclerc**

France

**John Tayu Lee**

UK

**Rebecca Lee**

USA

**Andrew Chee Keng Lee**

UK

**Chih-Hsin Lee**

Taiwan

**amanda Lee**

Australia

**Miryoung Lee**

USA

**Eun-Young Lee**

Canada

**Hae-Young Lee**

South Korea

**Kiyoung Lee**

South Korea

**Paul Lee**

Hong Kong

**Albert Lee**

Hong Kong

**Janet Lee**

USA

**Boung Lee**

Korea

**Paul Lee**

Hong Kong

**Eun Lee**

Korea

**Burkhard Leeb**

Austria

**Pattara Leelahavarong**

Thailand

**Amnesty LeFevre**

USA

**Mengistu Legesse**

Ethiopia

**Branka Legetic**

USA

**Håkan Leifman**

Sweden

**Margus Lember**

Estonia

**Ana Leme**

Brazil

**Seblewengel Lemma**

Ethiopia

**Maud Lemoine**

UK

**Elli Leontsini**

USA

**Anja Leppin**

Denmark

**Felice Le-Scherban**

USA

**Catherine Lesko**

USA

**Doris Leung**

Hong Kong

**Ying Ying Leung**

Singapore

**Jill Levenson**

USA

**Richard Levy**

USA

**Susanna Lewerin**

UK

**Kiara Lewis**

UK

**Ryan Lewis**

USA

**Els Leye**

Belgium

**Shugang Li**

China

**Haochu Li**

China

**Hao Li**

China

**Bai Li**

UK

**Jian Li**

Germany

**S Li**

China

**Shengxu Li**

USA

**Jian Li**

Germany

**Kaigang Li**

USA

**Ying Liang**

China

**Yajun Liang**

Sweden

**Shan Liao**

Canada

**Yue Liao**

USA

**Shih-Cheng Liao**

Taiwan

**Arve Lie**

Norway

**Alan Lifson**

USA

**Karen Liller**

UK

**Pao-Hwa Lin**

USA

**Chunqing Lin**

USA

**Jutta Lindert**

Germany

**Martin Lindstrom**

Sweden

**Kristina Lindvall**

Sweden

**Jennifer Linebarger**

USA

**Lisa Ling**

China

**Giuseppe Liotta**

Italy

**Yiing Mei Liou**

Taiwan

**Katherine Lippel**

Canada

**JF Lisón**

Spain

**Alyson Littman**

USA

**Chris Liu**

USA

**Qiyong Liu**

China

**Xiaona Liu**

Netherlands

**George Liu**

Australia

**Yu Liu**

USA

**Xiaodong Liu**

USA

**Yuewei Liu**

China

**Lei Liu**

USA

**Charles Livingstone**

Australia

**Scott Lloyd**

UK

**Sara Lodi**

USA

**Adrian Loerbroks**

Germany

**Christopher A. Loffredo**

USA

**Eric Lofgren**

USA

**Monica Löfvander**

Sweden

**Siew Yim Loh**

Malaysia

**Eskindir Loha**

Ethiopia

**Maria Lohan**

UK

**Lurdes Lomba**

Portugal

**Zané Lombard**

South Africa

**Richard Long**

Canada

**Jo Longman**

Australia

**T Longvah**

India

**Stefan Lönnberg**

Norway

**Pier Luigi Lopalco**

Sweden

**Luís Lopes**

Portugal

**Theo Lorenc**

UK

**Natalie Lorent**

Belgium

**Lucia Maria Lotrean**

Romania

**Penny Love**

Australia

**Marian Loveday**

South Africa

**Melanie Lowe**

Australia

**Tina M. Lowrey**

France

**Wenhua Lu**

USA

**Zuxun Lu**

China

**Chang-qin Lu**

China

**Tsung-Hsueh Lu**

Taiwan

**Yanxia Lu**

Singapore

**Yuan Lu**

USA

**Yoel Lubell**

Thailand

**Sean Lucan**

USA

**Robyn Lucas**

Australia

**Alessandra Lugo**

Italy

**Kai L**

China

**Karoline Lukaschek**

Germany

**Julie Lumeng**

USA

**Juan de Dios Luna del Castillo**

Spain

**Crick Lund**

South Africa

**Rikke Lund**

Denmark

**Feijun Luo**

USA

**Xianghua Luo**

USA

**Winnie Luseno**

USA

**Katherine Lust**

USA

**Katherine Lust**

USA

**Marc Luy**

Austria

**Ida Luzzi**

Italy

**Kate Lyden**

USA

**Marita Lynagh**

Australia

**Caroline Lynch**

UK

**Brigid Lynch**

Australia

**Wenjie Ma**

USA

**Jun Ma**

China

**Guansheng Ma**

China

**Gary Maartens**

South Africa

**Ruth Mabry**

Oman

**Sara Macdonald**

UK

**Mark Macek**

USA

**Daiane Machado**

Brazil

**Aristides Machado-Rodrigues**

Portugal

**Shingai Machingaidze**

South Africa

**Ethel Maciel**

Brazil

**James Macinko**

USA

**Diane Mack**

Canada

**Natasha Mack**

USA

**Kelly Mackenzie**

UK

**Roger Mackett**

UK

**Freya MacMillan**

Australia

**Jon Macy**

USA

**Mohsen Maddah**

Iran

**Raglan Maddox**

Canada

**Alberto Madeiro**

Brazil

**Sphiwe Madiba**

South Africa

**Claire Madigan**

Australia

**Ida E. H. Madsen**

Denmark

**Andreas Maercker**

Switzerland

**Martijn Maessen**

Netherlands

**Anthea Magarey**

Australia

**Zhila Maghbooli**

Iran

**Robert Magnani**

Indonesia

**Nicola Magnavita**

Italy

**Roger Magnusson**

Australia

**Catherine L. Mah**

Canada

**Amir Abbas Mahabadi**

Germany

**Jagadish Mahanta**

India

**Tanmay Mahapatra**

India

**Jaclyn Maher**

USA

**Mohamed Mahfouz**

Sudan

**Shahid Mahmood**

Pakistan

**Emily Mailey**

USA

**Olivier Maillard**

Mayotte

**John Mair-Jenkins**

UK

**Shannon Majowicz**

Canada

**Donna Mak**

Australia

**Peter Makaula**

Malawi

**Tomi Mäkinen**

Finland

**Kristen Malecki**

USA

**Rahul Malhotra**

Singapore

**Shelly Malhotra**

USA

**Kimberley Mallan**

Australia

**Mats Malqvist**

Singapore

**Alessia Mammone**

Italy

**Hadii Mamudu**

USA

**Milena Adina Man**

Romania

**Mark Manary**

USA

**Semira Manaseki-Holland**

UK

**Samuel Manda**

South Africa

**Bidisha Mandal**

USA

**Richard Mann**

UK

**Fredrik Mansson**

Sweden

**Karen Manzo**

USA

**Aimei Mao**

Macao

**Linda Marc**

USA

**Andrea Marcellusi**

Italy

**Sebastià March**

Spain

**Dirce Marchioni**

Brazil

**Ulrich Marcus**

Germany

**Johanna Maree**

South Africa

**Katherine Margo**

USA

**Carla Marienfeld**

USA

**Edmore Marinda**

South Africa

**Elisa Marques**

Portugal

**Letícia Marques dos Santos**

Brazil

**Elaine Marqueze**

Brazil

**Loraine Marrett**

Canada

**Roger Marshall**

New Zealand

**Ineke Marshall**

USA

**Brandon Marshall**

USA

**Elise Marsicano**

France

**Joachim Marti**

UK

**Emily Martin**

USA

**Averria Martin**

USA

**Mary Martinasek**

USA

**Jennifer Martin-Biggers**

USA

**Fred Martineau**

UK

**Domenico Martinelli**

Italy

**Iván Martínez-Baz**

Spain

**Miguel Martín-Matillas**

Spain

**Sandra Martins**

Portugal

**Koutatsu Maruyama**

Japan

**Miesha Marzell**

USA

**Joanna Maselko**

USA

**Linnet Masese**

USA

**Saidur Mashreky**

Bangladesh

**John Mason**

USA

**Deborah Masquio**

Brazil

**Peter Massey**

Australia

**Ruth Masterson Creber**

USA

**Colin Mathers**

Switzerland

**Annabel Matheson**

Australia

**Suzanna Mathew**

UK

**Anne Mathews**

USA

**Catherine Mathews**

South Africa

**Kaaren Mathias**

India

**Marie-Eve Mathieu**

Canada

**Joseph Matovu**

Uganda

**Tandi Edith Matsha**

South Africa

**Alberto Matteelli**

Italy

**Lynn Matthews**

USA

**Calum Mattocks**

UK

**Rapeephan Maude**

Thailand

**Massimo Maurici**

Italy

**Daniel Mauss**

Germany

**Rooyen Mavenyengwa**

Zimbabwe

**Taro Mawatari**

Japan

**Claire Mawditt**

UK

**Colleen Maxwell**

Canada

**Linda May**

USA

**Lindsay Mayberry**

USA

**Roy William Mayega**

Uganda

**Ahmed Yacoob Mayet**

Saudi Arabia

**Brandy Maynard**

USA

**Andreas Mayr**

Germany

**Jacob Mazalale**

Malawi

**Humphrey Mazigo**

Tanzania

**Sigal Mazor**

Israel

**Salig Mazta**

India

**Gerald Mazurek**

USA

**Anthony Mbonye**

Uganda

**John McAlaney**

UK

**Debra McCallum**

USA

**Heather McCauley**

USA

**Laura McCloskey**

USA

**Elaine McColl**

UK

**Megan Ann McCrory**

USA

**Marjorie McCullagh**

USA

**Patricia McDaniel**

USA

**Scott McDonald**

Netherlands

**Sheila McDonald**

Canada

**Scott McDonald**

UK

**Noreen McDonald**

USA

**Peggy McDonough**

Canada

**Scott McEwen**

Canada

**Jonathan McGavock**

UK

**Claire McGoldrick**

UK

**Pat McGovern**

USA

**Gerald McGwin**

USA

**Scott McIntosh**

USA

**Kerry McIver**

USA

**Kathy McKay**

Australia

**Martin McKee**

UK

**Karma Mckelvey**

USA

**Suzanne McKenzie**

Australia

**Lyle McKinnon**

South Africa

**Lyle McKinnon**

South Africa

**Rachael Mclean**

New Zealand

**Jane McLeod**

USA

**Philip McLoone**

UK

**Robert McMillen**

USA

**Candace McNaughton**

USA

**Jessica Mcneil**

Canada

**Geraldine McNeill**

UK

**Amy McPherson**

Canada

**Susan McRoy**

USA

**Sanjay Mehta**

USA

**Rohini Mehta**

USA

**Laurenz Meier**

Switzerland

**Raul Mejia**

Argentina

**Sumiko Mekaru**

USA

**Hans Olav Melberg**

Norway

**Nadine Melhem**

USA

**Nada Melhem**

Lebanon

**Jaymie Meliker**

USA

**Ann Melvin**

USA

**Glenn Melvin**

Australia

**Emily Mendenhall**

USA

**Amy Mendenhall**

USA

**Shanthi Mendis**

Switzerland

**Jacqueline Menezes**

Brazil

**Ritesh Menezes**

Saudi Arabia

**Linghui Meng**

China

**Xiaojun Meng**

China

**Allison Meni**

USA

**Giovanni Mento**

Italy

**Nancy Menzel**

USA

**Stewart Mercer**

UK

**Lisa Merlo**

USA

**Joav Merrick**

Israel

**Arthur Mesas**

Spain

**Gabriele Messina**

Italy

**Carla Meurk**

Australia

**Jaimie Meyer**

USA

**Beth Meyerson**

USA

**Anna Meyer-Weitz**

South Africa

**Aliapsha Meysamie**

Iran

**Rafael Meza**

USA

**Gabor Mezei**

USA

**Nathalie Michels**

Belgium

**Susan Michie**

UK

**Jonathan Micieli**

Canada

**Lisa Micklesfield**

South Africa

**Keren Middelkoop**

South Africa

**Ewa Mierzejewska**

Poland

**Massimo Miglioretti**

USA

**Lene Mikkelsen**

Australia

**Kathryn Millar**

USA

**Tim Millar**

UK

**Lynne Millar**

Australia

**Kathleen Miller**

USA

**Alexander Millman**

USA

**Erik Millstone**

UK

**Allison Milner**

Australia

**Jacqueline Milton**

USA

**Abul Milton**

Australia

**Leia Minaker**

Canada

**A. Minas**

USA

**Irina Mindlis**

USA

**Karl Minges**

USA

**Stephen James Minton**

Ireland

**Emanuele Miraglia Del Giudice**

Italy

**Claudio Miranda**

Brazil

**Andrew Mirelman**

USA

**Marta Miret**

Spain

**Dawn Misra**

USA

**Benjamin Missbach**

Austria

**Josef Mitas**

Czech Republic

**Jonathan Mitchell**

USA

**Soma Mitra**

Malaysia

**Joanna Mitri**

USA

**Eva Mitrová**

Slovakia

**Kyoko Miura**

Australia

**Paul Mkandawire**

Canada

**Abdallah Mkopi**

Tanzania

**Blandina Theophil Mmbaga**

Norway

**Bruno Mmbando**

Tanzania

**George Mnatzaganian**

Australia

**Amy Mobley**

USA

**Naomi Modeste**

USA

**Mette Mogensen**

Denmark

**Yasmin Mohamed**

Australia

**Mohamed Izham Mohamed Ibrahim**

Malaysia

**Ghorbanali Mohammadi**

Iran

**Abolfazl Mohammadi**

Iran

**Vikram Mohan**

Malaysia

**Viswanathan Mohan**

India

**Mohammadreza Mohebbi**

Austria

**Ralph Möhler**

Germany

**Mary Kate Mohlman**

USA

**Rahim Moineddin**

Canada

**Anu Molarius**

Sweden

**Javier Molina-Garcia**

Spain

**Gerard Molloy**

Ireland

**Sandra Momper**

USA

**Joaquin Moncho**

Spain

**Alison Mondul**

USA

**Joan Monin**

USA

**Evelyn Marielle Monninkhof**

Netherlands

**Courtney Monroe**

USA

**Ute Mons**

Germany

**Carl Montague**

South Africa

**Ali Montazeri**

Iran

**Rob Moodie**

Australia

**Gabriel Moore**

Australia

**Melinda Moore**

USA

**Alison Moore**

USA

**Catrin Moore**

UK

**Zohar Mor**

Israel

**Jose Moraes**

Brazil

**Carla Moreira**

Portugal

**Geraldine Moreno-Black**

USA

**Angelo Moretto**

Italy

**Gemma Morgan**

UK

**Alison Morgan**

Australia

**Michela Jane Angela Morleo**

UK

**Olga Morozova**

USA

**Christopher Morrison**

USA

**Leanne Morrison**

UK

**Todd Morrison**

Canada

**Jesper Mortensen**

Denmark

**Kevin Mortimer**

UK

**Katie Morton**

UK

**Giuseppe Moscelli**

UK

**Kathleen Moser**

USA

**Hans-Joachim Mosler**

Switzerland

**Sarah Moss**

South Africa

**Varvara Mouchtouri**

Greece

**Farah Mouhanna**

Lebanon

**Erly Moura**

Brazil

**Foong Ming Moy**

Malaysia

**Anne Moyer**

USA

**Elias Mpofu**

Australia

**Anna Mueller**

USA

**Evi Muggli**

Australia

**L Mughini-Gras**

Netherlands

**Wilson Winstons Muhwezi**

Uganda

**Oscar J. Mujica**

USA

**Andrew Mujugira**

Uganda

**Dick Mul**

Netherlands

**Katherine Muldoon**

Canada

**William Muller**

USA

**Caroline Mulvaney**

UK

**Colleen Munoz**

USA

**Daniel Murdock**

USA

**Harvey Murff**

USA

**Baba Musa**

Nigeria

**Beate Muschalla**

Germany

**Dara Musher-Eizenman**

USA

**Geofrey Musinguzi**

Uganda

**Adamson Muula**

Malawi

**Mercy Mvundura**

USA

**Henry Mwambi**

South Africa

**Puja Myles**

UK

**Jessica Gall Myrick**

USA

**KariKalan N**

India

**Kirsten Nabe-Nielsen**

Denmark

**Juliet Nabyonga-Orem**

Uganda

**Abhijit Nadkarni**

UK

**Masato Nagai**

Japan

**Eiji Nagayasu**

Japan

**Gera E Nagelhout**

Netherlands

**Rebekah Nagler**

USA

**Q Nahar**

Bangladesh

**Joanne Naidoo**

South Africa

**Pren Naidoo**

South Africa

**Eknath Naik**

USA

**Cho Naing**

Malaysia

**Suchithra Naish**

Australia

**Mehdi Najafzadeh**

USA

**Georgina Nakafero**

UK

**Miharu Nakanishi**

Japan

**Tonja Nansel**

USA

**Nicola Nante**

Iran

**Lucy Napper**

USA

**Sharon Nappier**

USA

**Muazzam Nasrullah**

Germany

**Lifoter Navti**

Cameroon

**Ajay Nayak**

USA

**Patti-Jean Naylor**

Canada

**Sarah Nechuta**

USA

**Saharnaz Nedjat**

Iran

**Roni Neff**

USA

**Joel Negin**

Australia

**Georges Nemer**

Lebanon

**Cory Neudorf**

Canada

**Linda Neuhauser**

USA

**Anne Neumann**

Germany

**Yehuda Neumark**

Israel

**Stephen Neville**

New Zealand

**Morkor Newman Owiredu**

Zimbabwe

**Robert Newton**

USA

**David Neyens**

USA

**Frida Ngalesoni**

Norway

**Mbong Eta Ngole**

Cameroon

**Quynh Nguyen**

USA

**Cattram Nguyen**

Australia

**Alexandra Nicholson**

UK

**Mary Nicolaou**

Netherlands

**Loredana Nicoletti**

Italy

**David Niebuhr**

USA

**Claire Niedzwiedz**

UK

**Karen Nieuwenhuijsen**

Netherlands

**AA Nikolskii**

Russia

**Per Nilsen**

Sweden

**Kerstin Nilsson**

Sweden

**Andreas Nilsson**

Sweden

**Sanjoy Ningthoujam**

India

**Midori Nishiyama**

Japan

**Catherine Nixon**

UK

**Christiana Noestlinger**

Belgium

**Masayuki Noguchi**

Japan

**Eva Nohlert**

Sweden

**Hanna Maria Nohynek**

Finland

**Andreu Nolasco**

Spain

**Helene Nordahl**

Denmark

**Lena Nordgren**

Sweden

**Cheryl Nordstrom**

USA

**Paul Norman**

UK

**Thor Norstrom**

Sweden

**Kate Northstone**

UK

**Chiara Nosarti**

UK

**Jean Jacques Noubiap**

Cameroon

**Dario Novak**

Croatia

**Jacob Novignon**

Ghana

**Thomas Novotny**

USA

**Mary Patricia Nowalk**

USA

**Paulina Nowicka**

Sweden

**Krzysztof Nowosielski**

Poland

**Elaine Nsoesie**

USA

**Robert Ntozini**

Zimbabwe

**John Nuckols**

USA

**Fred Nuwaha**

Uganda

**Jennifer Nuzzo**

USA

**George Nyandoro**

Zimbabwe

**Alan Nyitray**

USA

**Deborah Oakley**

USA

**Richard Oberhelman**

USA

**Chikaodili Obidike**

USA

**Joseph Obure**

Tanzania

**Rhoune Ochako**

Kenya

**Jessica Ochalek**

USA

**Hirotaka Ochiai**

Japan

**Caroline Ochieng**

Sweden

**Catherine C O'Connor**

Australia

**Meredith O'Connor**

Australia

**Bridianne O'Dea**

Australia

**Wilson Odero**

Kenya

**Angela Odoms-Young**

USA

**Grainne ODonoghue**

Ireland

**Olumuyiwa Odusanya**

Nigeria

**Willie Oglesby**

USA

**Maria Ogrzewalska**

Brazil

**Juhwan OH**

South Korea

**Kelly Lemos Ohara**

Portugal

**Blythe O'Hara**

Australia

**Britt Elin Øiestad**

Norway

**OE Ojo**

Nigeria

**Jerry Okal**

Kenya

**Scott Okamoto**

USA

**K Okanurak**

Thailand

**David Okech**

USA

**Chizimuzo Okoli**

USA

**Onyebuchi Okosieme**

UK

**Pinar Okyay**

Turkey

**Beatrice Olack**

Kenya

**Doina Olaru**

Australia

**Olaniyi Olayinka**

USA

**Michael Oldham**

USA

**ELizabeth Oliveras**

Mozambique

**Lemessa Oljira**

Ethiopia

**Kate O'Loughlin**

Australia

**Samuel Olowookere**

Nigeria

**Olufemi Oloyede**

Nigeria

**Jay Olshansky**

USA

**Tina Olsson**

Sweden

**Pia Olsson**

Sweden

**Dana Lee Olstad**

Australia

**Bukola Olutola**

South Africa

**Ian Olver**

Australia

**Nasrin Omidvar**

Iran

**Carol O'Neil**

USA

**Jennifer O'Neill**

USA

**Philip O'Neill**

UK

**Elijah Onsomu**

USA

**Oluwakare Opaneye**

USA

**Treena Orchard**

Canada

**Eyal Oren**

USA

**Ana Claudia Ornelas**

Brazil

**Nosakhare Orobaton**

USA

**Norm O'Rourke**

Canada

**Alicia Orta-Ramirez**

USA

**Adriana Ortega**

Malaysia

**Livia Elisa Ortensi**

Italy

**Derick Nii Mensah Osakunor**

Ghana

**Karen Osilla**

USA

**Malina Osman**

Malaysia

**Richard Thomas Oster**

Canada

**Ståle Østhus**

Norway

**Tracey O'Sullivan**

Canada

**Therese O'Sullivan**

Australia

**Atsuhiko Ota**

Japan

**Alfonso Otero Gonzalez**

Spain

**Nasih Othman**

Iraq

**Samuel Oti**

Kenya

**David Otiashvili**

Georgia

**Suzie Otto**

Netherlands

**Suh-Ruu Ou**

USA

**Karen Oude Hengel**

Netherlands

**Samiratou Ouédraogo**

UK

**Dennis Ougrin**

UK

**Marja-Leena Ovaskainen**

Finland

**Simon Overland**

Norway

**Onikepe Owolabi**

UK

**Ellis Owusu-Dabo**

Ghana

**Omar Oyarzabal**

USA

**Adewale Oyeyemi**

Nigeria

**Sachiko Ozawa**

USA

**Nilufer Ozaydin**

Turkey

**Barbara Pacelli**

Italy

**Cristina Padez**

Portugal

**Bijaya Padhi**

India

**Marcello Pagano**

USA

**Charlotte Page**

USA

**Franco Pagnoni**

Switzerland

**Anjali Pai**

UK

**Ian Painter**

USA

**Subhamoy Pal**

USA

**Cristina Palacios**

Puerto Rico

**Domingo Palacios-Ceña**

Spain

**Joseph Palamar**

USA

**Charles Palenik**

USA

**Tia Palermo**

Italy

**Miranda Pallan**

UK

**Stephen Palmer**

UK

**William Pan**

USA

**Demosthenes Panagiotakos**

Greece

**Amita Pandey**

India

**Massimiliano Panella**

Italy

**Raina Pang**

USA

**Pinaki Panigrahi**

USA

**Alexandra Pankova**

Czech Republic

**Supasit Pannarunothai**

Thailand

**Annalisa Pantosti**

Italy

**Maria Papadakaki**

Greece

**Vassiliki Papaevangelou**

Greece

**Dimitrios Papagiannis**

Greece

**Stavroula Papapdodima**

Greece

**Athanasia Papazafiropoulou**

Greece

**Kristine Pape**

Norway

**Marguerite Pappaioanou**

USA

**Marie-Claude Paquette**

Canada

**Raveen Parboosing**

South Africa

**Sanjoti Parekh**

Australia

**Yong-Moon Park**

USA

**Sohyun Park**

USA

**David Parker**

USA

**Katharine Parkes**

UK

**Alison Parkes**

UK

**Suchitra Parkhad**

India

**Anne-Maree Parrish**

Australia

**Roberto Pasetto**

Italy

**Pauline Paterson**

UK

**James Paturas**

USA

**Francisco Paumgartten**

Brazil

**Toby Pavey**

Australia

**Patricia Pavlinac**

USA

**Milena Pavlova**

Netherlands

**Valerie Paz Soldan**

Peru

**Elizabeth Peach**

Australia

**Rebecca Pearl**

USA

**Natalie Pearson**

UK

**Fiona Pearson**

UK

**Sallie Pearson**

Australia

**Anne Peasey**

UK

**Robert Peck**

Tanzania

**Corinne Peek-Asa**

USA

**Nasheeta Peer**

South Africa

**Ivica Pelivan**

Croatia

**Christine Pellegrini**

USA

**Jennifer Pelletier**

USA

**Fiona Pelly**

Australia

**Karl Peltzer**

Thailand

**Peter Pemberton-Ross**

Switzerland

**Wenceslao Penate**

Spain

**Eva Penelo**

Spain

**Yingwei Peng**

Canada

**Xiaoxia Peng**

China

**Amy Pennay**

Australia

**Tarra Penney**

UK

**Louisa Peralta**

Australia

**Monique Pereboom**

Netherlands

**Telmo Pereira**

Portugal

**Adriana Perez**

USA

**Carolina Perez Ferrer**

UK

**Carlos Pérez García-Pando**

USA

**Napoleon Perez-Farinos**

Spain

**Carolina Perez-Ferrer**

UK

**Henry Perry**

USA

**Yael Perry**

Australia

**Janey Peterson**

USA

**Brennan Peterson**

USA

**Audrey Petit**

France

**Miruna Petrescu-Prahova**

USA

**Dennis Petrie**

Australia

**Kelley Pettee Gabriel**

USA

**Stefano Petti**

Italy

**Audrey Pettifor**

USA

**John Pettifor**

South Africa

**Patrizio Pezzotti**

Italy

**Alena M. Pfeil**

Switzerland

**Minh Pham**

Australia

**Benjamin Phelps**

USA

**Rune Philemon**

Tanzania

**Carl Phillips**

Canada

**Lisa Phillips**

UK

**Julie Phillips**

USA

**Karen Phillips**

Canada

**David Phillips**

USA

**Thomas Piasecki**

USA

**Ken Pidd**

Australia

**Matthias Pierce**

UK

**Iris Pigeot**

Germany

**David Pigott**

UK

**Kathleen Pike**

USA

**Richard Pilsner**

USA

**Heather Pines**

USA

**Joaquim Pinheiro**

USA

**Melissa Pinto**

USA

**Filipa Pinto-Ribeiro**

Portugal

**Jillian Pintye**

USA

**Roberta Pirastu**

Italy

**Pedro Pisa**

South Africa

**Francesco Pistelli**

Italy

**Sabrina Pit**

Australia

**Eileen Pitpitan**

USA

**Virginia Pitzer**

USA

**Sandra Plachta-Danielzik**

Germany

**Martin Plöderl**

Austria

**David Plummer**

Australia

**May Ee Png**

Singapore

**Maurizio Pocchiari**

Italy

**Piero Poletti**

Italy

**Alessandro Polichetti**

Italy

**Keshia Pollack**

USA

**Christina Pollard**

Australia

**Laura Pomeroy**

USA

**Maurizio Pompili**

Italy

**Wouter Poortinga**

UK

**Frank Popham**

UK

**Jahan Porhomayon**

USA

**Aaron Porter**

USA

**Ann Post**

Sweden

**Paola Postacchini**

Italy

**Riq Pouget**

USA

**Elisabeth Poulet**

France

**Catherine Pound**

Canada

**Farshad Pourmalek**

Canada

**Mieneke Pouwelse**

Netherlands

**Thomas Power**

USA

**John Powles**

UK

**Rosa Prato**

Italy

**Albert Presto**

USA

**John Preston**

UK

**Kay Price**

Australia

**Patricia Priest**

New Zealand

**Diana Prieto**

USA

**Lucia Prihodova**

Ireland

**Stefany Primeaux**

USA

**James Prior**

UK

**Antonio Prista**

Mozambique

**Karin Proper**

Netherlands

**Joanne Protheroe**

UK

**Wendy Prudhomme O'Meara**

Kenya

**Christy Pu**

Taiwan

**Anna Puig-Ribera**

Spain

**Carol Pullen**

USA

**Richard Pulsford**

UK

**Dewi Ismajani Puradiredja**

UK

**Pekka Puska**

Finland

**Nancy Puttkammer**

USA

**Glen Pyle**

Canada

**Qibin Qi**

USA

**Jin Qin**

USA

**Xiu Qiu**

China

**Gianluca Quaglio**

Italy

**Maira Quintanilha**

Canada

**Chantal Quinten**

Sweden

**Lisa Quintiliani**

USA

**Alex Quistberg**

USA

**Anuradha R**

India

**Miriam Rabkin**

USA

**Jerome Rachele**

Australia

**George Rachiotis**

Greece

**Jany Rademakers**

Netherlands

**Houshang Rafatpanah**

Iran

**Alberto Raggi**

Italy

**Malathi Raghavan**

Canada

**Azizur Rahman**

Australia

**Tanvi Rai**

UK

**Subhadra Rajanaidu**

UK

**Luis Rajmil**

Spain

**Ranjani Ramachandran**

India

**Amutha Ramadas**

Malaysia

**Anu Rammohan**

Australia

**Rosa Ramón-Bonache**

Spain

**Diana Rancourt**

USA

**Kathryn Rand**

USA

**Louise Randall**

Australia

**Janis Randall Simpson**

Canada

**V Ranjbar**

USA

**Geetha Ranmuthugala**

Australia

**Otso Rantonen**

Finland

**Chalapati Rao**

Australia

**Dennis Raphael**

Canada

**Kumanan Rasanathan**

USA

**Davide Rasella**

Brazil

**Sabrina Rasheed**

Bangladesh

**Abdur Rasheed**

Pakistan

**T Rashid**

USA

**Barbara Rath**

Germany

**Elena Ratschen**

UK

**Nancy Raymond**

USA

**Marissa Raymond-Flesch**

USA

**John Read**

Australia

**Sarah Reagan-Steiner**

USA

**Jennifer Reath**

Australia

**Nicola Reavley**

Australia

**Amanda Rebar**

Australia

**Peter Rebeiro**

USA

**Bernd Rechel**

UK

**Leanne Redman**

USA

**Mary Redmayne**

Australia

**Sean Redmond**

USA

**Vaughan Rees**

USA

**Aaron Reeves**

UK

**Enrique Regidor**

Spain

**K Regmi**

UK

**SA Régnier**

Switzerland

**Juergen Rehm**

Canada

**Colin Rehm**

USA

**Jürgen Rehm**

Canada

**Natasha Reid**

Australia

**Elizabeth Reifsnider**

USA

**Thomas Reischl**

USA

**Aiguo Ren**

China

**Tara Rendo-Urteaga**

Spain

**Donna Rennie**

Canada

**Andre Renzaho**

Australia

**Jenna Reps**

UK

**Patricio Retamal**

Chile

**Megan Reynolds**

USA

**Giovanni Rezza**

Italy

**Ryan Rhodes**

Canada

**Mary Rice**

USA

**Vesta Richardson**

Mexico

**Iman Ridda**

Australia

**Julie Riddell**

UK

**Nicola Ridgers**

Australia

**Nathaniel Riggs**

USA

**Arja Rimpelä**

Finland

**Giancarlo Ripabelli**

Italy

**Matthew Ritchey**

USA

**Joseph Ritter**

USA

**Evangelos Rizos**

Greece

**Caterina Rizzo**

Italy

**Ubaidur Rob**

Bangladesh

**Lorraine Robbins**

USA

**Rachel Roberts**

Australia

**Lucy Robertson**

Norway

**Jennifer Robertson-Wilson**

Canada

**Lisa Robinson**

USA

**Eric Robinson**

UK

**Dylan Roby**

USA

**Jessica Rodrigues**

USA

**Carlos Rodriguez**

USA

**Lindsey M. Rodriguez**

USA

**Rosa Rodriguez-Monguio**

USA

**Darrin Rogers**

USA

**Anne Elizabeth Rogers**

UK

**David Rojas-Rueda**

Spain

**Italia Rolle**

USA

**Roberto Romi**

Italy

**Sarah Rominski**

USA

**Amber Ronteltap**

Netherlands

**Rimante Ronto**

Australia

**Robin Room**

Australia

**Eva Roos**

Finland

**Elisabeth Root**

USA

**Annina Ropponen**

Finland

**Rafaela Rosário**

Portugal

**Michael Rosato**

UK

**Chad Rose**

USA

**Sydney Rosen**

USA

**Mark Rosenberg**

Canada

**Dori Rosenberg**

USA

**Philip Rosenberg**

USA

**Paul Rosile**

USA

**Bernard Rosner**

USA

**Kathleen Rospenda**

USA

**Kathryn Ross**

USA

**Perran Ross**

Australia

**Alyssia Rossetto**

Australia

**Linda Rothman**

Canada

**Alexandra Rouquette**

France

**Ash Routen**

UK

**Marion Rowland**

Ireland

**Sam Rowlands**

UK

**Louise Rowling**

Australia

**Jason Roy**

USA

**Cathy Rozmus**

USA

**Adolfo Rubinstein**

Argentina

**Rima Rudd**

USA

**Anne Caroline Rudisill**

UK

**Paul Rukavina**

USA

**Rosemary Rushmer**

UK

**Ginny Russell**

UK

**Elizeus Rutebemberwa**

Uganda

**Geert Rutten**

Netherlands

**A Rychwalska**

USA

**Agnieszka Rychwalska**

Poland

**Eric S. Drollette**

USA

**Charumathi Sabanayagam**

Singapore

**Kalpana Sabapathy**

UK

**Deborah Saber**

USA

**Chiara Sabina**

USA

**Paul Sacco**

USA

**Cheryl Sadowski**

Canada

**Tani Sagna**

Burkina Faso

**Rihlat Said-Mohamed**

South Africa

**Michaela Saisana**

Italy

**Eiko Saito**

Japan

**Nagahito Saito**

Japan

**Akihiko Saitoh**

Japan

**Usha Salagame**

Australia

**Rehana Salam**

Pakistan

**Christopher Salas-Wright**

USA

**Miguel Salinero-Fort**

Spain

**Amanda Salis**

Australia

**Matthew Salois**

USA

**Haroon Saloojee**

South Africa

**Emanuel Salvador**

Brazil

**Taraz Samandari**

USA

**Oddrun Samdal**

Norway

**Andriy V. Samokhvalov**

Canada

**T.Alafia Samuels**

Barbados

**Zila Sanchez**

Brazil

**Jesus Sanchez**

USA

**Gavin Sandercock**

UK

**Rachel Sandford**

UK

**G Sandstrom**

Sweden

**Zawadi Sanga**

Tanzania

**Carine Sangaleti**

Brazil

**Alba Santaliestra-Pasias**

Spain

**D Santamaria**

USA

**Silvina Santana**

Portugal

**Paula Clara Santos**

Portugal

**Maria Paula Santos**

Portugal

**Diana Santos**

Portugal

**Ailiana Santosa**

Sweden

**Gloria Santos-Beneit**

Spain

**Adriana Sanudo**

Brazil

**Martin Alfredo Canaan Sapag Aucar**

Argentina

**Valeria Saraceni**

Brazil

**Mary Saridi**

Greece

**Emanuel Sarinho**

Brazil

**Lisonkova Sarka**

Canada

**Shah-Jalal Sarker**

UK

**Malabika Sarker**

Bangladesh

**Gloria E. Sarto**

USA

**Kurt Sartorius**

South Africa

**Benn Sartorius**

South Africa

**Sheela Sathyanarayana**

USA

**Julie Saunders**

Australia

**Travis Saunders**

Canada

**Adem Sav**

Australia

**Naomi Saville**

UK

**Elena Savoia**

USA

**Andrew J. Saxon**

USA

**Kourosh Sayehmiri**

Iran

**Sunil Sazawal**

USA

**Rebecca Schacht**

USA

**Stanley J. Schaffer**

USA

**Dena Schanzer**

Canada

**Enid Schatz**

USA

**Frieder Schaumburg**

Germany

**Vanessa Schick**

USA

**Barbara Schillo**

USA

**Jasper Schipperijn**

Denmark

**GM Schippers**

Netherlands

**Ketra Schmitt**

Canada

**Carol Schmitt**

USA

**Peter-Ernst Schnabel**

Germany

**Amy Schneeberg**

Canada

**Klaus Schneider**

Germany

**Alexandra Schneider**

Germany

**Stephanie Schoeppe**

Australia

**Shaun Scholes**

UK

**Kirsty Scholes-Balog**

Australia

**Michael Schomaker**

South Africa

**Nadja Schott**

Germany

**Evert G Schouten**

Niue

**Roel Schouteten**

Netherlands

**James B Schreiber**

USA

**Jan Schugk**

Finland

**Alyssa Schultz**

USA

**Julian Schulze zur Wiesch**

Germany

**John Schuna**

USA

**Sheree Schwartz**

USA

**Robert Schwartz**

Canada

**Anne-Maree Schwarz**

Solomon Islands

**Jürgen Schweikart**

Germany

**Jane Scott**

Australia

**Whitney Scott**

UK

**Paul Scott**

USA

**Bridie Scott-Parker**

Australia

**Mark Scudder**

USA

**André Seabra**

Portugal

**Jamie Seabrook**

Canada

**David Seal**

USA

**Holly Seale**

Australia

**Sarah Emma Seaton**

UK

**Emerson Sebastiao**

USA

**Anne Sebert Kuhlmann**

USA

**Janet Seeley**

Uganda

**Jan Seghers**

Belgium

**Victor Segura-Jimenez**

Spain

**Jorma Seitsamo**

Finland

**Chloe Sellwood**

UK

**Linda Selvey**

Australia

**Sonia Semenic**

Canada

**Katherine Semrau**

USA

**Pallav Sengupta**

India

**Paramita Sengupta**

India

**Theresa Senn**

USA

**Carlo Senore**

Italy

**Teresa Senserrick**

Australia

**Teresa Senserrick**

Australia

**Tetine Sentell**

USA

**Diego Serraino**

Italy

**Ruchan Sertoz**

Turkey

**Sam-Ang Seubsman**

Thailand

**Anna Sevcikova**

Czech Republic

**Carlo Severini**

Italy

**Kashif Shafique**

Pakistan

**Mohammad Saad Shaheed**

USA

**Jorida Shahinas**

Albania

**Saeid Shahraz**

USA

**Masoud Shamaei**

Iran

**Marian Shanahan**

Australia

**Kwame Shanaube**

Zimbabwe

**Carrie Shandra**

USA

**Ce Shang**

USA

**Aparna Shankar**

UK

**Bhavani Shankar**

UK

**Frank Shann**

Australia

**Harry Shannon**

Canada

**Eugene Shapiro**

USA

**Saida Sharapova**

USA

**Mamak Shariat**

Iran

**Alyssa Sharkey**

USA

**Vartika Sharma**

India

**Vinita Sharma**

Nepal

**Phyllis Sharps**

USA

**Lisa Sharwood**

Australia

**Benjamin Shaw**

USA

**Jill Shawe**

UK

**Bettina Shell-Duncan**

USA

**Rachel Shelton**

USA

**Chan Shen**

USA

**Sheela Shenoi**

USA

**Shweta Shenoy**

India

**Vicky Sheppeard**

Australia

**Roya Sherafat**

USA

**Recinda Sherman**

USA

**Zumin Shi**

Australia

**Qiyun Shi**

Canada

**Simon Shibli**

UK

**Rahul Shidhaye**

India

**Christopher Shiels**

USA

**Yusuke Shimakawa**

France

**Scott Shimotsu**

USA

**Shoji Shinkai**

Japan

**Tetyana Shippee**

USA

**Eric Shiroma**

USA

**Abigail Shoben**

USA

**David Shoham**

USA

**Camille Short**

Australia

**Mohamed Shoukri**

Saudi Arabia

**Sadeep Shrestha**

USA

**Nipun Shrestha**

Nepal

**Sadeep Shrestha**

USA

**Rosalyn Shute**

Australia

**Drissa Sia**

Canada

**Spyros Siakavellas**

Greece

**Kamran Siddiqi**

UK

**Juned Siddique**

USA

**Martin Siegel**

Germany

**Karen Siegel**

USA

**Aaron Siegler**

USA

**Maria Jose Sierra**

Spain

**Jose Sierrra**

Spain

**John Sieverdes**

USA

**Erik Sigmund**

Czech Republic

**Alessio Signorini**

USA

**Asia Sikora Kessler**

USA

**Luis Silva**

Cuba

**Kelly Silva**

Brazil

**Paula Silva**

Portugal

**Analiza Silva**

Portugal

**Maria Laura Silva**

France

**Michael Silverberg**

USA

**Debra Simmons**

USA

**Kate Simms**

Australia

**Dorien Simons**

Belgium

**Jacob Simonsen**

Denmark

**Alexander Simpson**

Canada

**AS Singh**

Netherlands

**Tushar Singh**

USA

**Rosalyn Singleton**

USA

**Chutima Sirikulchayanonta**

Thailand

**Chesmal Siriwardhana**

Sri Lanka

**Chesmal Siriwardhana**

Sri Lanka

**Susan Sisson**

USA

**Cindy Sit**

Hong Kong

**Anna Siukola**

Finland

**Ylva Skaner**

Sweden

**Eva Skillgate**

Sweden

**Marit Skogstad**

Norway

**Amy Slater**

UK

**James Slaughter**

USA

**Terry Slevin**

Australia

**Zofia A. Slonska**

Poland

**Judith Sluiter**

Netherlands

**Sandra Small**

Canada

**Nada Smigic**

Serbia

**Catherine Smith**

New Zealand

**Mark Smith**

Canada

**Ben Smith**

Australia

**Catherine Smith**

USA

**Jordan Smith**

Australia

**Katherine Smith**

USA

**Rachel Smith**

USA

**Nathan Smith**

USA

**Stella Smith**

Nigeria

**Ben Smith**

Australia

**N.R. Smith**

UK

**Jennifer Smith-Merry**

Australia

**Miguel A Sogorb**

Spain

**Orielle Solar Hormazabal**

Chile

**Berge Solberg**

Norway

**Maria Giuliana Solinas**

Italy

**Svetlana Solovieva**

Finland

**Joonmo Son**

Singapore

**Samir Soneji**

USA

**Lixin Song**

USA

**In Han Song**

South Korea

**Qing-Kun Song**

China

**Thomas Songer**

USA

**Suneeta Soni**

UK

**Kristine Sorensen**

Netherlands

**Alberto Soriano-Maldonado**

Spain

**Ali Soroush**

Iran

**Erica Sosa**

USA

**Giovanni Sotgiu**

Italy

**Carla A. Sousa**

Portugal

**Ulla Sovio**

UK

**Kaan Sozmen**

Turkey

**Enea Spada**

Italy

**Fredrik Spak**

Sweden

**Ross Sparks**

Australia

**Dean Spears**

India

**June Spector**

USA

**Katherine Speirs**

USA

**Ilene Speizer**

USA

**Lisa Spencer**

Australia

**Stefanie Sperlich**

Germany

**Niko Speybroeck**

Belgium

**Elodie Speyer**

USA

**Juliane Spiegler**

Germany

**Jana Spilkova**

Czech Republic

**Bruno Spire**

France

**Jorinde Spook**

USA

**Michele Spoont**

USA

**Sandra Springer**

USA

**Andrew Springer**

USA

**Jane Springett**

Canada

**Jorge Srabstein**

USA

**Chandrashekhar Sreeramareddy**

Nepal

**K Srithanaviboonchai**

USA

**Gideon St.Helen**

USA

**Stella Stabouli**

Greece

**Diane Stadler**

USA

**Amanda Staiano**

USA

**Roger Stancliffe**

Australia

**Debbi Stanistreet**

UK

**Maja Stanojevic**

Serbia and Montenegro

**Ben Stansfield**

UK

**Jerod Stapleton**

USA

**Kaspar Staub**

Switzerland

**Matthew Stefanak**

USA

**Michael Stefanone**

USA

**Aryeh Stein**

USA

**Riley Steiner**

USA

**Robert Steinglass**

USA

**Peter Steinmann**

Switzerland

**Eva Steliarova-Foucher**

France

**Irina Stepanov**

USA

**Elena Sterdt**

Germany

**Vicky Stergiopoulos**

Canada

**Christopher Stevenson**

Australia

**Anna Stewart Ibarra**

USA

**Jim Stewart-Evans**

UK

**Nelia Steyn**

South Africa

**David Stieb**

Canada

**Sigrid Stjernswärd**

Sweden

**Emily Stockings**

Australia

**Heidi Stöckl**

UK

**Barbara Stoecker**

USA

**Michal Stoklosa**

USA

**Jodi Stookey**

USA

**Thomas Stopka**

USA

**Carla Storr**

USA

**Michael Stoto**

USA

**Rebecca Stotzer**

USA

**Bjørn Heine Strand**

Norway

**Andrea Streng**

Germany

**Nanette Stroebele-Benschop**

Germany

**Claudia Strugnell**

Australia

**Ellen Struijk**

Netherlands

**Laura Struik**

Canada

**Helen Struthers**

South Africa

**Pierluigi Struzzo**

Italy

**James Stuart**

UK

**Bjarte Stubhaug**

Norway

**Dasa Stupica**

Slovenia

**Pedro G Suarez**

USA

**Ramnath Subbaraman**

India

**Malavika Subramanyam**

India

**Madhuri Sudan**

USA

**Brian Suffoletto**

USA

**Takehiro Sugiyama**

Japan

**Takemi Sugiyama**

Australia

**Deeptha Sukumar**

USA

**Patrick Sullivan**

USA

**Shahnaz Sultan**

USA

**Ernest Sumaili**

Congo, Democratic Republic

**Khurana Sumeeta**

India

**Yingxian Sun**

China

**Zilin Sun**

China

**Sakari Bertel Alfred Suominen**

Finland

**Inoka Suraweera**

Sri Lanka

**Fernanda G C Surita**

Brazil

**Stefan Sütterlin**

Norway

**Kohta Suzuki**

Japan

**Erik Svendsen**

USA

**Johan Svensson**

Sweden

**James Swartz**

USA

**Lance Swenson**

USA

**Ilse Swinkels**

Netherlands

**Bryan Sykes**

USA

**Ian Symonds**

Australia

**Beata Szostakowska**

Poland

**Richard Szydlo**

UK

**Mieczyslaw Szyszkowicz**

Canada

**Daniel Taber**

USA

**Angela Taft**

Australia

**Silvio Tafuri**

Italy

**Richard Tahtinen**

Iceland

**Jiro Takaki**

Japan

**Esa-Pekka Takala**

Finland

**Tadashi Takeshima**

Japan

**Yin On Yvonne Tam**

USA

**Jamie Tam**

USA

**Kosuke Tamura**

USA

**Shinsuke Tanaka**

USA

**Stephanie Tanamas**

Australia

**Weiming Tang**

China

**Alice Tang**

USA

**Ryutaro Tanizaki**

Japan

**Yelena Tarasenko**

USA

**Valerie Tarasuk**

Canada

**Shema Tariq**

UK

**Marie-Pierre Tavolacci**

France

**Louis Tay**

USA

**Jennifer Taylor**

USA

**Anne Taylor**

Australia

**John Taylor**

UK

**Brandie Taylor**

USA

**Adrian Taylor**

UK

**Tatiana Taylor Salisbury**

UK

**Michel Tchuenche**

Tanzania

**Saskia te Velde**

Netherlands

**Nathan Tefft**

USA

**Norman Temple**

Canada

**David Templeton**

Australia

**Gill Ten Hoor**

Netherlands

**Tiew-Hwa Katherine Teng**

Australia

**Beverly Tepper**

USA

**Masaru Teramoto**

USA

**Benedetto Terracini**

Italy

**Laura Terragni**

Norway

**Paul Terry**

USA

**Kay Teschke**

Canada

**Nai-peng Tey**

Malaysia

**Megan Teychenne**

Australia

**Chrissie Thakwalakwa**

Malawi

**Montarat Thavorncharoensap**

Thailand

**Katherine Theall**

USA

**Hla-Hla Thein**

Canada

**Marie-José Theunissen**

Netherlands

**Cecilie Thogersen-Ntoumani**

Australia

**Emma Thomas**

UK

**Diana Thomas**

USA

**Scott Thomas**

Canada

**Roger Thomas**

Canada

**Barbara Thomlison**

USA

**Shirley Thompson**

Canada

**Janice Thompson**

UK

**Michael Thompson**

USA

**Carol B Thompson**

USA

**Lisa Thompson**

USA

**Janice Thompson**

UK

**Rebecca Thomson**

Australia

**George Thomson**

New Zealand

**Maria Thomson**

USA

**Ingibjörg Thorisdottir**

Iceland

**Kevan Thorley**

UK

**Simon Thornley**

New Zealand

**Lukar Thornton**

Australia

**Maree Thorpe**

Australia

**Sannie Vester Thorsen**

Denmark

**Einar Thorsteinsson**

Australia

**Katherine Thurber**

Australia

**James Tielsch**

USA

**Hong Van Tieu**

USA

**David Timberlake**

USA

**Neil Tippett**

UK

**Michele Tizzoni**

Italy

**Shahnaz Tofangchiha**

Iran

**Susanna Toivanen**

Sweden

**Maria Toledo**

Chile

**Eleni Tolma**

USA

**Ghasem (Sam) Toloo**

Australia

**Brian Tom**

UK

**G Scalia Tomba**

Italy

**Elena Tomba**

Italy

**Connie Tompkins**

USA

**Tanja Tomson**

Sweden

**Martin Tondel**

Sweden

**Olukemi Tongo**

Nigeria

**Aleksandra Torbica**

Italy

**Stefania Toselli**

Italy

**Ellen Toth**

Canada

**Vivian Towe**

USA

**Alberto Tozzi**

Italy

**Bich Tran**

Australia

**Thach Tran**

Vietnam

**Thanh Tam Tran**

Australia

**Catherine Travers**

Australia

**Justin Trogdon**

USA

**Cecilia Trucchi**

Italy

**Gabriel Trueba**

Ecuador

**Faustin Pascal Tsague Manfo**

Cameroon

**Jennifer Tsai**

USA

**Jack Tsai**

USA

**Jaw-Shiun Tsai**

Taiwan

**Sandro Tsang**

Hong Kong

**Kim Tsao**

USA

**Tshokey Tshokey**

Bhutan

**Cleon Tsimbos**

Greece

**Janice Tsoh**

USA

**Kelvin Tsoi**

Hong Kong

**Lungiswa Tsolekile**

South Africa

**Kenji Tsunoda**

Japan

**Andrew Tu**

Canada

**Terry Tudor**

UK

**Josep A. Tur**

Spain

**Monique Turner**

USA

**Katrina Turner**

UK

**Michelle Turner**

Canada

**Gavin Turrell**

Australia

**Torill Helene Tveito**

Norway

**Brooke Tvermoes**

USA

**Laurie Twells**

Canada

**Konstantinos Tziomalos**

Greece

**Valerie Ubbes**

USA

**Tomoko Udo**

USA

**Cesar Ugarte-Gil**

Peru

**Julia Uhanova**

Canada

**Léonie Uijtdewilligen**

Singapore

**Zia Ul Haq**

UK

**Shahid Ullah**

Australia

**Bernhard Ultsch**

Germany

**M. Renee Umstattd Meyer**

USA

**Leanne Unicomb**

Bangladesh

**Noortje Uphoff**

UK

**Lianne Urada**

USA

**Mark Urassa**

Tanzania

**Michael Ussher**

UK

**Anneli Uusküla**

Estonia

**Mandana Vahabi**

Canada

**Abhinav Vaidya**

Nepal

**Rodolfo Valdez**

USA

**Rupa Valdez**

USA

**Maria Valente**

Brazil

**João Valente-dos-Santos**

Portugal

**Jeff Vallance**

Canada

**Helen Vallianatos**

Canada

**Kirsten Vallmuur**

Australia

**Patricia Van Assema**

Netherlands

**Ed van Beeck**

Netherlands

**Birgit van Benthem**

Netherlands

**Jantien van Berkel**

Netherlands

**Jelle Van Cauwenberg**

Belgium

**Tom H Van De Belt**

Netherlands

**T Van De Laar**

Netherlands

**Bart Van Den Borne**

Netherlands

**Ingrid Viola Francine Van Den Broek**

Netherlands

**Johan Hubert André Van Der Heyden**

Belgium

**Klazine Van Der Horst**

Switzerland

**Sander Van Der Linden**

USA

**Marianne A.B. Van Der Sande**

Netherlands

**Nickie Van Der Wulp**

Netherlands

**Bernardus Van Der Zeijst**

Netherlands

**Dane Van Domelen**

USA

**Job Van Exel**

Netherlands

**Veerle Van Holle**

Belgium

**Dave Van Kann**

Netherlands

**Wendy Van Lippevelde**

Belgium

**Trevor Van Mielro**

Canada

**Mark Van Ommeren**

Switzerland

**Mireille Van Poppel**

Netherlands

**Eline H. Van Roekel**

Netherlands

**Heidi Van Rooyen**

South Africa

**Johannes Van Rooyen**

South Africa

**Lenie Van Rossem**

Netherlands

**Ronan Van Rossem**

Belgium

**Arjella Van Scheppingen**

Netherlands

**Edwin Van Teijlingen**

UK

**Corneel Vandelanotte**

Australia

**Leigh Vanderloo**

Canada

**Stefanie Vandevijvere**

New Zealand

**Alain Vandormael**

South Africa

**Anna H van't Hoog**

Netherlands

**Veronica Varela Mato**

UK

**Maria Ines Varela-Silva**

UK

**Joanna Vearey**

South Africa

**Tord Finne Vedoy**

Norway

**Ruut Veenhoven**

Netherlands

**Lennert Veerman**

Australia

**Jenny Veitch**

Australia

**Raman Velayudhan**

Switzerland

**Dr.Anoop Velayudhan**

India

**Sukumar Vellakkal**

India

**Sudhir Venkatesan**

UK

**Soren Ventegodt**

Djibouti

**Giulietta Venturi**

Italy

**Jan Verbakel**

Belgium

**Willem Verberk**

Netherlands

**Pierre Verger**

France

**Linda Verhoef**

Netherlands

**Maite Verloigne**

Belgium

**Aditi Verma**

India

**Heleen Vermandere**

Belgium

**Evelien Vermeulen-Smit**

Netherlands

**Michel Vernay**

France

**Christopher Verrico**

USA

**Eleni Verykouki**

Greece

**Fenicia Vescio**

Italy

**Taryn Vian**

USA

**Fernanda Vidigal**

Brazil

**N Vielot**

USA

**Andreas Vilhelmsson**

Sweden

**Emilio Villa-González**

Ecuador

**Livia Villar**

Brazil

**Kerri Viney**

Australia

**Carmen Beatriz Visioli**

Italy

**Barbara Vizmanos**

Mexico

**Hristina Vlajinac**

Serbia and Montenegro

**Bruce Vogel**

USA

**Salim Vohra**

UK

**Rachel Volberg**

USA

**Antonio Volpi**

Italy

**Mikaela von Bonsdorff**

Finland

**Arne von Delft**

South Africa

**Sophie Von Stumm**

UK

**Sirenda Vong**

Cambodia

**Carmen Voogt**

Netherlands

**Wieger Voskuijl**

Netherlands

**Christine Voss**

Canada

**Nicholas Vozoris**

Canada

**Alpo Vuorio**

Finland

**Jennifer Wagman**

USA

**Verina Waights**

UK

**Minako Wakasugi**

Japan

**Simon Walker**

UK

**Tim Walker**

Rwanda

**Martin Wall**

New Zealand

**NIna Wallerstein**

USA

**Ken Wallston**

USA

**Adam Walsh**

Australia

**Sophie Walsh**

Israel

**Zach Walsh**

Canada

**Judd Walson**

USA

**Angela Walter**

USA

**Kathryn Walton**

Canada

**Richard Wamai**

USA

**Xia Wan**

China

**Handan Wand**

Australia

**Bonnie Wandera**

Uganda

**Haibo Wang**

China

**Yujie Wang**

USA

**Rui Wang**

USA

**Yuan-Pang Wang**

Brazil

**Man Ping Wang**

Hong Kong

**Yujie Wang**

USA

**Harry Hao-Xiang Wang**

UK

**Xiaohua Wang**

China

**Yu-Chun Wang**

Taiwan

**Man Ping Wang**

Hong Kong

**Shuang Wang**

USA

**Haibo Wang**

China

**Harry Hao-Xiang Wang**

UK

**JianLi Wang**

Canada

**Xuewen Wang**

USA

**Yong Wang**

China

**L Wang**

Canada

**Yu Wang**

China

**Haibo Wang**

China

**Shaoming Wang**

China

**Harry Hao-Xiang Wang**

China

**Rhoda Wanyenze**

Uganda

**Tony Ward**

USA

**Dianne Ward**

USA

**Paul Ward**

Australia

**Catherine Ward**

South Africa

**Vicky Ward**

UK

**Catharine Ward Thompson**

UK

**James Ware**

USA

**Saman Warnakulasuriya**

UK

**Joshua Warren**

USA

**Shaneda Warren Andersen**

USA

**Sharada Wasti**

Nepal

**Rosanna Watowicz**

USA

**Angela Watson**

Australia

**Jude Watson**

UK

**Amber Watts**

USA

**Geeke Waverijn**

Netherlands

**Sonali Wayal**

UK

**Andrea Waylen**

UK

**Andrea Waylen**

UK

**Michael Weaver**

USA

**Kate Webber**

Australia

**Jonathon Webber**

New Zealand

**Jacqui Webster**

Australia

**E Kipling Webster**

USA

**Niels Wedderkopp**

Denmark

**Lara Weinstein**

USA

**Thomas Weiss**

USA

**William Weiss**

USA

**James R. Welch**

Brazil

**Berhe Weldearegawi**

Ethiopia

**Greg Welk**

USA

**Robert Wellman**

USA

**Yvonne Wells**

USA

**Eden Wells**

USA

**Li Ming Wen**

Australia

**Tzai-Hung Wen**

Taiwan

**Anna Lena Wennberg**

Sweden

**Heini Wennman**

Finland

**Erik L. Werner**

Norway

**Sonia Wesche**

Canada

**Lisa Wexler**

USA

**Simone Weyers**

Germany

**Joan Wharf Higgins**

Canada

**Ana Wheelock**

UK

**James White**

UK

**Daniel White**

USA

**Laura White**

USA

**Joanna White**

New Zealand

**Jennifer Whitehill**

USA

**Nicole Whitehurst**

USA

**Geoffrey Whitfield**

USA

**Richard Whitley**

USA

**Maxine Whittaker**

Australia

**Priya Wickramaratne**

USA

**Micael Widerström**

Sweden

**Frank Wieber**

Germany

**Mark Wieland**

USA

**Monika Wijeratne**

USA

**Sonja Wilhelm Stanis**

USA

**Anna Wilkinson**

Australia

**Katalin Wilkinson**

South Africa

**Marc C. Willemsen**

Netherlands

**Joshua Willey**

USA

**Craig Williams**

Australia

**Sue Williams**

Australia

**Gemma Williams**

UK

**Nathalie Williams**

USA

**Sue Williams**

Australia

**Brie Williams**

USA

**Quintin Williams Jr**

USA

**Amanda Willig**

USA

**Micky Willmott**

UK

**Jessica Willoughby**

USA

**Caroline Wilson**

UK

**Sacoby Wilson**

USA

**Andrew Wilson**

Australia

**Erin Wilson**

USA

**Helen Winefield**

Australia

**Marcy Winget**

USA

**Aliza Wingo**

USA

**Pattanee Winichagoon**

Thailand

**Mirko Winkler**

Switzerland

**Sandra Winter**

USA

**Aprilianto Wiria**

Netherlands

**Norman Wirsik**

Germany

**Andrew Wister**

Canada

**Charles Witzel**

UK

**Eileen Willis**

Australia

**Mirkuzie Woldie**

Ethiopia

**Michael Wolfe**

USA

**Mary Wolff**

USA

**Shane Shucheng Wong**

USA

**Jessica Wong**

Hong Kong

**Man Yee, Emmy Wong**

Hong Kong

**Mee Lian Wong**

Singapore

**Zhi Wong**

Malaysia

**Darryl Wood**

USA

**James Woodall**

UK

**Catherine Woods**

Ireland

**Sarah Woolf-King**

USA

**Alemayehu Worku Yalew**

Ethiopia

**Adugna Woyessa**

Ethiopia

**Darren Wraith**

Australia

**Dominika Wranik**

Canada

**Ricardo Wray**

USA

**Laura Wray-Lake**

USA

**Jim Wright**

UK

**Charlotte Wright**

UK

**David Bin-Chia Wu**

Taiwan

**Chia-Yi Wu**

Taiwan

**Qi Wu**

UK

**David Wu Bin-Chia**

Malaysia

**Gwenllian Wynne-Jones**

UK

**Xiaoling Xiang**

USA

**Huiyun Xiang**

USA

**Shunqing Xu**

China

**Fuzhong Xue**

China

**Gautam Yadama**

USA

**Dhananjay Yadav**

India

**Prashant Yadav**

Spain

**Bereket Yakob**

Ethiopia

**Tamaki Yamada**

Japan

**Tiê Parma Yamato**

Australia

**Takashi Yamauchi**

Japan

**Yongping Yan**

China

**Lijing Yan**

USA

**Guiyun Yan**

USA

**Xueling Yang**

China

**Marie Yap**

Australia

**Ohid Yaqub**

UK

**Lucy Yardley**

UK

**Ilya Yaroslavsky**

USA

**Dingwei Ye**

China

**Hung-Wen Yeh**

USA

**Adoke Yeka**

Uganda

**Laurann Yen**

Australia

**Hye-Ah Yeom**

South Korea

**Barbaros Yet**

UK

**Edwina Yeung**

USA

**Albert Yeung**

UK

**John Yfantopoulos**

Greece

**Solomon A Yimer**

Norway

**Peng Yin**

China

**Xinhua Yin**

China

**Oded Yitschaky**

Israel

**Renata Yokota**

Belgium

**Koji Yonemoto**

Japan

**Naohiro Yonemoto**

Japan

**Hua Yong**

Australia

**Mieko Yoshihama**

USA

**Philippa Youl**

Australia

**April Young**

USA

**Charles Young**

South Africa

**Katherine Young**

USA

**Jim Young**

Switzerland

**Jeanine Young**

Australia

**Kimberly Yousey-HIndes**

USA

**Zhiping Yu**

USA

**Jiao Yumei**

China

**Fakir Yunus**

Bangladesh

**Mauro Zaccarelli**

Italy

**Syed Sohail Zahoor Zaidi**

Pakistan

**Joanna Zajkowska**

Poland

**Lijana Zaletel-Kragelj**

Slovenia

**Nickolas Zaller**

USA

**Margareth Zanchetta**

Canada

**Faika Zanjani**

USA

**Roger Zapata**

Peru

**Gustavo Zarini**

USA

**Dorota Zarnowiecki**

Australia

**Gianguglielmo Zehender**

Italy

**Yohannes Zenebe**

Ethiopia

**Temesgen Zewotir**

South Africa

**Chunliu Zhan**

USA

**Weihai Zhan**

USA

**Yiqiang Zhan**

Sweden

**Jie Zhang**

USA

**Bing Zhang**

China

**Xiu-Jun Zhang**

China

**Wei Zhang**

USA

**ZhiJie Zhang**

China

**Xiaochu Zhang**

China

**Zhu-Ming Zhang**

USA

**Qiang Zhang**

China

**SK Zhang**

China

**Li Zhang**

China

**Tiejun Zhang**

China

**Yuejen Zhao**

Australia

**Yun Zhao**

Australia

**Nan Zhao**

USA

**Pinpin Zheng**

China

**Tao Zheng**

China

**Tiantian Zheng**

USA

**Yibiao Zhou**

China

**Yeyi Zhu**

USA

**Jinliang Zhu**

Denmark

**Motao Zhu**

USA

**Haidong Zhu**

USA

**Xun Zhuang**

China

**Guihua Zhuang**

China

**Nicolas Ziebarth**

USA

**Frederick Zimmerman**

USA

**Frank Zimmermann-Viehoff**

Germany

**Walter Zingg**

Switzerland

**Vadim Zipunnikov**

USA

**Julie Zito**

USA

**Farzaneh Zolala**

Iran

**Carmen Zorrilla**

Puerto Rico

**Diana Zuckerman**

USA

**Isaac Zulu**

USA

**Maria Clelia Zurlo**

Italy

**Yvonne Zurynski**

Australia

**Cosmas Zyaambo**

Zambia

**Sa'Ed Zyoud**

Palestinian Territory, Occupied

